# Language Structures May Adapt to the Sociolinguistic Environment, but It Matters What and How You Count: A Typological Study of Verbal and Nominal Complexity

**DOI:** 10.3389/fpsyg.2018.01141

**Published:** 2018-08-14

**Authors:** Kaius Sinnemäki, Francesca Di Garbo

**Affiliations:** ^1^Department of Languages, University of Helsinki, Helsinki, Finland; ^2^Department of Linguistics, Stockholm University, Stockholm, Sweden

**Keywords:** inflectional synthesis, grammatical gender, language complexity, population size, second language learning, sociolinguistic environment, language typology, adaptation

## Abstract

In this article we evaluate claims that language structure adapts to sociolinguistic environment. We present the results of two typological case studies examining the effects of the number of native (=L1) speakers and the proportion of adult second language (=L2) learners on language structure. Data from more than 300 languages suggest that testing the effect of population size and proportion of adult L2 learners on features of verbal and nominal complexity produces conflicting results on different grammatical features. The results show that verbal inflectional synthesis adapts to the sociolinguistic environment but the number of genders does not. The results also suggest that modeling population size together with proportion of L2 improves model fit compared to modeling them independently of one another. We thus argue that surveying population size alone may be insufficient to detect possible adaptation of linguistic structure to the sociolinguistic environment. Rather, other features, such as proportion of L2 speakers, prestige and social network density, should be studied, and if demographic numeric data are used, they should not be used in isolation but rather in competition with other sociolinguistic features. We also suggest that not all types of language structures within a given grammatical domain are equally sensitive to the effect of sociolinguistic variables, and that more exploratory studies are needed before we can arrive at a reliable set of grammatical features that may be potentially most (and least) adaptive to social structures.

## 1. Introduction

Recent research suggests that linguistic structures adapt to the sociocultural environment in which languages are spoken (Ladd et al., 2015). Since languages are acquired and used in different social contexts, those contexts may bias acquisition and usage: linguistic structures become adapted to these social niches and this, over time, may be reflected in typological distributions (Lupyan and Dale, 2010; Sinnemäki, 2014). Central ideas in this approach have been:

- Small communities with dense social networks, few adult learners, and a great deal of shared knowledge favor linguistic structures that are difficult for adults (e.g., irregularity in inflectional paradigms);
- Large communities with loose social networks, more adult learners, and less shared knowledge favor more regular and easier to learn linguistic structures (e.g., regularity in inflectional paradigms, transparency; Trudgill, 2011b).

These ideas have also been tested empirically, with focus on the relationship between language complexity and community size. However, the results have been conflicting. For instance, the number of cases seems to correlate inversely with the number of native speakers (Lupyan and Dale, 2010), but according to Bentz and Winter (2013) it correlates only with the proportion of L2 speakers in the community, and not with overall community size.

In this paper, we review a number of studies on language complexity, population size, and linguistic adaptation and contrast these findings with two empirical studies of our own. Study 1 focuses on verbal inflectional synthesis and Study 2 on grammatical gender. We take these features as instances of morphological complexity in the verbal and nominal domain, respectively. With respect to verbal inflectional synthesis, we find that only the number of L1 speakers has a significant effect on verbal complexity when the sociolinguistic features are modeled independently of one another. We also find that the proportion of L2 speakers has a significant effect on verbal complexity when modeled together with the number of L1 speakers in one and the same model. This suggests that the features may conspire in shaping language structure. With respect to grammatical gender, we find no significant effect of the sociolinguistic variables under study on the number of gender distinctions, both when the demographic predictors are considered independently and when they are modeled together. We also observe a confounding effect of data coding structure on the patterns detected by our models.

We highlight the discrepancies between the results and discuss the factors that could motivate them. Moreover, we argue that in order to establish more solid results on linguistic adaptation, demographic features must be studied in competition with each other and further combined with in depth studies of sociolinguistic and sociohistorical profiles. We also suggest that not all variables that describe crosslinguistic variation in a given domain of grammar may be equally suited to investigate how and whether this domain adapts to sociolinguistic structures. Selecting the right typological variables to test adaptive responses of language structures to social structures is thus crucial to studies in this field and requires going beyond existing typological databases.

## 2. Background

One of the main tenets of functional-typological linguistics is the idea that language structures are shaped by properties of human cognition as well as by the dynamics of social interaction (Beckner et al., 2009). The mechanisms by which languages adapt to their contexts of use are also considered to be the driving force of language variation and change (Givón, 2009; Bybee, 2010; von Mengden and Coussé, 2014). During the last decades a new trend of studies has developed within the language sciences, which tests these assumptions empirically by investigating the relationship between typological, sociolinguistic, and environmental variables based both on micro-level qualitative investigations (Kusters, 2003; DeLancey, 2014), and large scale quantitative studies (e.g., Lupyan and Dale, 2010; Dediu and Cysouw, 2013; Everett et al., 2015; for more references, see the review by Ladd et al., 2015). Phonemic inventory size, tone, degree of inflectional synthesis, inflectional morphology, lexical diversity, and lexical stability are some of the domains of language variation investigated so far within this approach, and in connection with an array of sociolinguistic and environmental factors such as population size, proportion of L2 speakers, number of neighboring languages, and humidity. In this paper, we study linguistic adaptation from the perspective of one domain of linguistic variation, morphological complexity, as measured through verbal inflectional synthesis and number of grammatical gender distinctions. We explore typological variation in these areas of grammar in relationship with demographic data on first language (=L1) and second language (=L2) speakers. We first introduce the grammatical phenomena under investigation. We then review a number of studies that have looked at the interactions between these features and the sociolinguistic variables under study.

### 2.1. Morphological complexity and verbal inflectional synthesis

Morphological complexity, taken as a measure of the degree of grammatical elaboration and internal structuring of words, has traditionally attracted much attention in typology. Since the nineteenth century languages were classified in three holistic morphological types: isolating (or analytic), fusional (or inflective), and agglutinative. It was believed that one parameter of typological variation, morphology, had predictive scope on the overall appearance of entire languages. This one-dimensional, holistic approach has been later rejected in typology, and, starting with the work of Sapir (1921), alternative classifications that break morphological typology into multiple and mutually interacting parameters have been proposed (see Plank, 1998, 1999, for more detailed review of the discussion). These more recent classifications cover multiple dimensions of variation, such as the internal complexity of the word (analytic vs. synthetic), the nature of morpheme boundaries (agglutination vs. fusion), and the extent to which several roots may be combined into one and the same word (incorporation).

In recent crosslinguistic research degree of inflectional synthesis has been especially a subject of interest. This label is used to refer to the number of morphemes or morphological categories that are realized in a word. Inflection is here defined as “those categories of morphology that are *regularly responsive to the grammatical environment* in which they are expressed” (Bickel and Nichols, 2007, p. 169). The main difference to derivation is that inflection is responsive to the grammatical (that is, morphological or syntactic) environment, whereas derivation is responsive to the lexical environment but not to the grammatical environment. For instance, in English the number of the subject is reflected in the morphological choices of agreement on the verb in sentences such as *the waiter likes ice cream* vs. *kids like ice cream*. In these examples agreement determines morphological choices based on the syntactic environment, whereas the choice of a derivational category, as in *waiter* vs. *waitress*, is entirely a lexical matter.

If a grammatical category, such as person, is expressed inflectionally as in the word *like-s*, the construction is said to be synthetic but if the category is expressed through a separate word, as in *will do*, the construction is said to be analytic (Bickel and Nichols, 2013).

In analytic constructions the relationship between the elements is syntactic and not morphological and the elements do not make up a grammatical word. It is well-known that grammatical and phonological criteria of wordhood do not coincide cross-linguistically (Dixon and Aikhenvald, 2002; Haspelmath, 2011). In the *AUTOTYP* database, which we use as our data source for verbal inflectional synthesis (see also section 3.2), this challenge has been solved by focusing on grammatical words. Synthesis is a matter of grammatical words but it is independent of phonological binding and therefore grammatical words can be composed of phonologically distinct words (Bickel and Nichols, 2007, 2013). The crucial issue here is that if a phonologically distinct word cannot be used alone without the verb and also in different orderings, then that word is part of the same grammatical word with the verb. Bickel and Nichols (2013) give the example of the tense marker *làay* in Hakha Lai (Tibeto-Burman). This marker is an independent phonological word as it bears tone and contains two moras, but it cannot be used independently of the verb and it always occurs in the same position relative to the verb, as in (1).


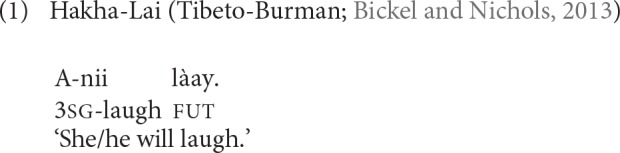


Together with the verb, the tense marker *làay* in Hakha Lai is an example of grammatical word.

The notion of word-level semantic density has also been used in the literature to refer to degree of inflectional synthesis (Bickel and Nichols, 2007, p. 188–193). Vietnamese is a language with very low semantic density of words, since words generally consist of only a single morpheme, as in (2). More toward the other end of the synthesis/semantic density scale are languages with very complex word structure, such as Turkish, illustrated in (3), which may attach up to ten or more inflectional and derivational morphemes into one and the same grammatical word.


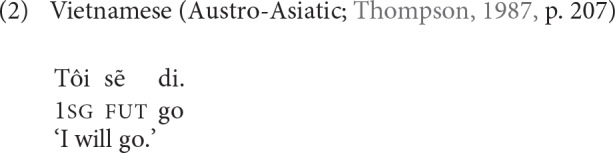



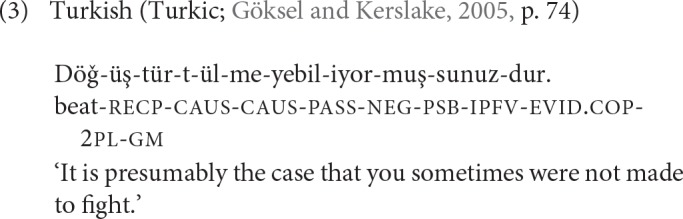


As shown in (3), morphological words in highly synthetic languages may sometimes correspond to a whole sentence in other languages.

Languages with a degree of inflectional synthesis comparable to Turkish are rather common around the world. Comparative data in the domain of verbal inflection suggests that almost half (44%; *n* = 145) of the world's languages have the same or higher degree of synthesis than Turkish (Bickel and Nichols, 2013). This distribution suggests that high word-internal complexity is not particularly difficult for children to acquire and for native speakers to use. Evidence from language acquisition supports this conclusion. By the age of two Turkish children fully master the nominal inflectional system and most of the verbal inflectional system as well (Slobin, 2005). Children also acquire inflectional cues equally or even faster than alternative cues, such as word order or prosody (Slobin and Bever, 1982). From the point of view of adult language use, high degree of synthesis should also pose no problems, whether in production or comprehension (see Kusters, 2003, p. 46–52 and references). However, compared to native speakers, adult learners are overall less sensitive to morphological structure during language processing in their L2 (Clahsen et al., 2010). Morphologically complex words have higher informational complexity and thus higher processing cost in word recognition (Moscoso del Prado Martín et al., 2004). Verbal inflection in particular poses major problems to adult learners but much less so to child learners (see Parodi et al., 2004, p. 670, and references there). This difficulty that adults have in learning and using complex inflection is related to a more general pattern supported by neurocognitive evidence: learning grammar in procedural memory creates more problems for adult learners than for L1 learners while acquiring lexical knowledge in declarative memory poses fewer such problems for adults (Ullman, 2005). This learning bias toward declarative memory means that adult learners prefer lexical strategies and periphrastic constructions over grammatical strategies, especially at low levels of exposure.

### 2.2. Morphological complexity and grammatical gender

Grammatical gender is one of the possible strategies that languages use to partition nouns into classes. Typically, these classifications may at least partially rest on semantic distinctions based on natural gender (as in the sex-based systems of the Romance languages), or on other parameters, such as animacy, size, or shape (as in the non-sex-based systems of the Bantu languages).

The most important definitional property of grammatical gender systems is that the encoding of grammaticalized classificatory distinctions is *displaced*. It does not only (or not necessarily) occur on nouns, but *must* appear on those words that are engaged in a syntactic relation with nouns. In languages with grammatical gender, attributive modifiers, predicates, and pronouns are the word classes that most typically carry gender marking through their inflectional morphology. The syntactic relation between nouns and carriers of gender marking is traditionally called *agreement*. Within typological literature, nouns are referred to as *controllers* of the agreement relationship because their gender controls the type of marking encoded through agreement. Conversely, those words whose inflectional morphology varies in agreement with the gender of a noun are labeled *targets* of the agreement relationship. Dahl (2004) regards grammatical gender as one of the most typical instances of *mature* grammatical phenomena in language: gender systems are long-lived features of language families and they usually presuppose intricate, non-trivial processes of grammaticalization.

In Italian (Indo-European, Romance) nouns are assigned to one of two genders: the masculine and the feminine. For at least a portion of the nominal lexicon (humans and higher animates), gender assignment is predicted based on sex. Displaced gender marking occurs on attributive modifiers, some of the pronouns, and past participles. Example (4) illustrates gender marking in Italian, both within and outside the noun phrase.


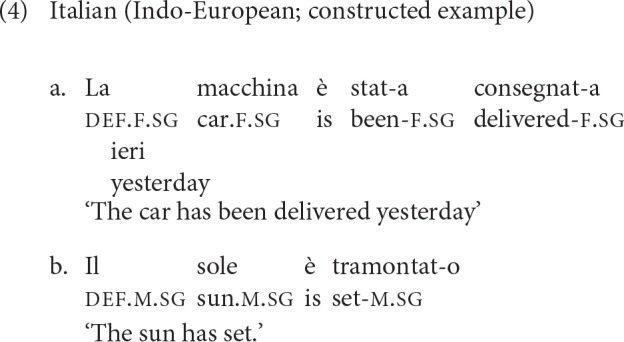


As is praxis within typology we use the label grammatical gender not just to refer to systems of noun classification of the Italian type, that is, based on natural gender and on two to three distinctions, but also to those systems that are typically found in many African and some Papuan languages, and that are often labeled *noun classes*. These systems may have up to almost 20 different agreement classes which are not always clearly motivated semantically. In Mufian (Torricelli; spoken in the East Sepik region of New Guinea), different suffixes on the noun and adjective as well as prefixes on the verb stand for different noun classes; Table 1 shows a selection of these.

**Table 1:** A selected set of noun classes in Mufian (Alungum et al., 1978, p. 93).

**Class**	**Example**	**Gloss**	**Noun suffix**	**Verb prefix**
	**singular/plural**		**singular/plural**	**singular/plural**
1	bol / bongof	“pig”	-l / -ngof	l- / f-
2	éngél / angof	“name”	-ngél / ngof	g- / f-
3	nalof / nalelef	“tooth”	-f / -lef	f- / f-
5	batéwin / batéwis	“child”	-n / -s	n- / s-
17	kos / kos	“course”	-s / -s	s- / s-

Grammatical gender, as defined above, can be associated with morphological complexity in two ways. Syntagmatically, gender marking is distributed over an utterance through agreement patterns, and several entities within that utterance may thus redundantly point to the gender of the controller noun. Paradigmatically, each word class that is sensitive to gender inflections typically displays as many forms as the number of genders to be distinguished. For instance, the Italian definite article has two forms distinguishing masculine and feminine gender both in the singular and in the plural (for a total of four distinct forms). In this paper, we do not look at these dimensions directly, but focus instead on the number of gender distinctions in a language. This is estimated based on the number of distinguishable agreement patterns, and thus at least indirectly relates to paradigmatic complexity, that is, to the number of subdistinctions available in a linguistic category (see Moravcsik and Wirth, 1986).

Corbett (2013a) identifies the presence of a gender system in 112 out of 257 sampled languages. The distribution of grammatical gender in the languages of his sample is rather skewed, both geographically and genealogically, which reflects an actual tendency in the overall distribution of gender systems. Gender systems are very common in some areas of the world, such as Africa and Eurasia, but rather rare in others, such as North America. This geographical bias is directly connected to a genealogical bias. The presence of grammatical gender is often a distinctive, stable feature of individual language families, whose members do usually also cluster geographically. Moreover, the presence of grammatical gender across language families is reinforced by areal contiguity. Even though geographically biased, the pervasive distribution of grammatical gender within individual language families and coherent linguistic areas suggests that under normal circumstances of language transmission gender systems are easily acquired and mastered by children and native speakers.

This is indeed confirmed in the literature. Studies of L1 acquisition of grammatical gender, focusing on different L1s and different types of gender systems, show that children are generally able to master at least aspects of the gender system of their native language by the age of three. They are usually better at relying on phonological rather than semantic cues for gender assignment, and the frequency of individual nouns in every-day speech affects how much they use a given gender marking pattern (for language specific studies of the acquisition of grammatical gender see, for instance, Suzman, 1980; Mulford, 1985; Mills, 1986; Desmuth, 2000; Eichler et al., 2013; Gagliardi and Lidz, 2014). Similarly, studies of language processing and comprehension show that gender marking plays an important role in processes of semantic and syntactic disambinguation in adult native speaker usage (see, for instance, Gunter and Friederici, 2000; Barber and Carreiras, 2005). Even though unproblematic in L1 acquisition and native speaker usage, grammatical gender is a challenge for non-native adult learners, and exactly for the same reasons that we mentioned in the case of verbal inflectional synthesis. Mastering gender marking presupposes the acquisition of complex patterns of inflection, which L2 speakers tend to struggle with, and thus to avoid^1^.

### 2.3. Does morphology adapt to social structure?

Processing difficulties that language users face are one of the driving factors behind language change if, following a usage-based approach to language, we assume that preferences in language use become conventionalized over time (e.g., Sinnemäki, 2014). It has been recently suggested that the processing difficulties that adults face in learning and using an L2 may end up having an effect on the (evolution of the) grammar of the native speakers as well (see e.g., Lupyan and Dale 2010; Bentz and Winter 2013; and references there). The magnitude of this effect crucially depends on the proportion of non-native speakers in the speech population. The larger the proportion of non-native speakers, the more their presence is likely to have an impact on the grammars of L1 users.

Maitz and Németh (2014) compare three types of German varieties against four indicators of morphosyntactic complexity (degree of synthesis being one of them), and to the effect that these varieties represent three distinct sociolinguistic and sociohistorical profiles: one highly standardized contact variety (Standard German), two high contact varieties (Kiche Duits and Unserdeutsch), and one low contact L1 variety (Cimbrian). The results show significant differences between the two types of high contact varieties, on the one hand, and the low contact L1 variety, on the other, with respect to all four parameters of morphosyntactic complexity. The impact of L2 learning on the evolution of ancient language varieties has been also studied. For instance, Skelton (2017) demonstrates that peculiar features of the Ancient Greek dialect of Pamphilia (at the phonological, morphological, syntactic, and lexical level) can be explained as the result of massive influence from Anatolian speakers, who represented the majority of the population in the area and spoke Greek as L2.

Verbal inflectional synthesis and grammatical gender have been shown to be sensitive to the effect of massive L2 learning. For instance, drawing on historical and contemporary data from Quechuan, Swahili, Arabic, and Scandinavian, Kusters (2003) shows that those language varieties which, throughout their history, were characterized by high proportions of adult non-native learners have simpler verbal morphology than their closest cognates with little or no history of exposure to non-native learners. Trudgill (1999, 2001) and McWhorter (2001, 2007) also argue that high contact language varieties, characterized by a significant increase in number of adult learners at some point throughout their history, are likely to lose grammatical gender. Examples of this would be, for instance, Persian, which has lost the gender system preserved by other Iranian languages, or many pidgin and creole languages, which tend to be devoid of grammatical gender irrespectively of the presence of this feature in their lexifiers and/or substrata. Similarly, Kusters (2003) also shows that gender agreement on verbs tends to simplify as a result of increased language contact. In all these cases loss of gender has been typically explained with the fact that gender marking is substantially afunctional from the point of view of effective communication and thus likely to be weakened/lost in non-native speaker usage. However, recent research by Blasi et al. (2017) on the dynamics of language transmission under creole emergence shows that creole languages do not exhibit any systematic structural simplification with respect to the two gender-related variables that the study accounts for, adjectival adnominal agreement and presence of gender distinctions on personal pronouns. Instead, both variables seem to be sensitive to ancestry, that is, they align with the structural type attested in either the lexifier or the substratum, and do not seem to be directly linked with the sociohistorical background the sampled languages share with other creoles. Whether some aspect of gender may adapt to sociolinguistic environment is thus a matter of current debate and open to exploration from different angles (see also section 3.3.3).

While research on linguistic adaptation in the domain of morphology has largely focused on non-native acquisition as a trigger of simplification (e.g., Kusters, 2003), evidence for the complexification of verbal morphology in the absence of large-scale non-native acquisition has also been provided. DeLancey (2014) showed that two Tibeto-Burman languages spoken in North East India, Boro (Boro-Garo branch of Tibeto-Burman) and Lai (Kuki-Chin branch of Tibeto-Burman) have different morphological profiles and are spoken in very different sociolinguistic environments. Boro, which has very little verbal morphology, is spoken by a large, widely distributed community in the Assam plains where there has historically been, and still is, much interaction with speakers of other languages. Lai, on the contrary, has developed new synthetic verbal morphology not present in proto-Tibeto-Burman and it is spoken in small relatively isolated hill communities in the mountain range which follows the India-Myanmar border. Trudgill (2017) argues that languages with polysynthetic morphology, that is, those with a very high ratio of morphemes per words and possibly also noun incorporation, tend to be spoken by small communities, with fewer than 10,000 speakers. These communities are also relatively isolated and have rather dense social network structure.

Recent quantitative typological research provides further evidence that population structure has an impact on language structures. Lupyan and Dale (2010) modeled the relationship between morphological complexity (measured on the basis of a set of 28 variables taken from the *World Atlas of Language Structures*), and the (log) number of native speakers with generalized linear modeling. In their study, speech community size was taken as a proxy for the degree of adult L2 learning in the community, under the assumption that languages with larger populations are more likely to engage in contact with other speech communities, and to be learned non-natively. The results of the study indicated that smaller languages tend to have higher degrees of morphological complexity than larger languages. This applied across geographical areas and language families, but also within language families. However, speech community size in itself is not the only predictor of change in language structures, and other sociolinguistic factors may need to be taken into account as well. This point has been made by Trudgill (2011a) in relation to phoneme inventory size and later empirically confirmed by Moran et al. (2012), who show that there is no statistical evidence for a correlation between phoneme inventory size and speech community size (see section 3.3.3).

While Lupyan and Dale (2010) used log number of speakers as a proxy for the degree of adult L2 learning in a given speech community, Bentz and Winter (2013) propose to evaluate the effects of adult L2 learning more directly, by taking into account the proportion of adult L2 learners in a given speech community (the speech community comprised of both native and non-native speakers) and assessing whether this has any effect on the number of grammaticalized case distinctions in a language. Although the sample used by Bentz and Winter (2013) is not particularly large (*n* = 66 languages), their data suggest that there is a strong inverse relationship between the number of cases and the proportion of adult non-native learners in the community: high proportion of adult non-native learners correlates with low number of cases and low proportion of adult non-native learners correlates with high number of cases. To emphasize the importance of measuring the proportion of non-native learners, they also show that, in their data set, population size (native + non-native speakers) has no effect on the number of cases (Bentz and Winter, 2013, p. 11).

In Study 1 we attempt to replicate the results of Lupyan and Dale (2010) by focusing on one dimension of their morphological complexity metric, notably the degree of inflectional synthesis on the verb. The data in their study is based on the chapter by Bickel and Nichols (2013) in *WALS*, which is in turn based on the *AUTOTYP* database. The original *AUTOTYP* data set contains a much more detailed analysis of inflectional synthesis than what was later included in *WALS*. The *WALS* format required authors to keep the number of levels limited for each variable and this means that variable levels are conflated in many chapters, including the one on verbal inflectional synthesis where, for instance, synthesis degrees 6 and 7 are conflated into one category “6-7.” This kind of conflation inevitably leads to loss of information, which we attempt to avoid in this paper by using the original and now expanded data of the *AUTOTYP* database (Bickel et al., 2017). The data set has information on inflectional synthesis in 309 languages. With respect to sociolinguistic variables, while, as mentioned above, Lupyan and Dale (2010) worked only with data on population size, in our study we consider both the number of L1 speakers as well as the proportions of L2 speakers. This choice of features models more closely the hypothesis put forward in sociolinguistic typology that the size and structure of a speech community, on the one hand, and the degree of language contact, on the other, should be taken into account simultaneously but also independently of each other (e.g., Trudgill, 2011a).

Dahl (unpublished) tests linguistic adaptation by looking at the relationship between the three *WALS* features devoted to grammatical gender^2^ and number of speakers. The results suggest that no consistent relationship can be found between any of the gender features and the number of speakers a language has (a weak positive correlation is however detected between non-sex-based gender systems and population data). In Study 2, we attempt to replicate these findings with a larger data set (*n* = 345). Differently from Dahl (unpublished) we focus only on one gender feature, the number of gender values, and consider only nominal gender, thus excluding pronominal gender systems, such as the one attested in English, from the data set. With respect to sociolinguistic variables, as in Study 1, we consider the log number of L1 speakers and the proportions of L2 speakers both in isolation and in combination with each other.

## 3. Typological case studies

We contrast the findings of the earlier research surveyed above with two empirical case studies of our own. The first study deals with the degree of inflectional synthesis on the verb, a common metric of complexity in cross-linguistic research (e.g., Kusters, 2003; Shosted, 2006; de Groot, 2008; Nichols, 2009; Kettunen, 2014). The second study deals with the number of grammatical genders in a language. Recent research regards the number of gender distinctions as one of the three main dimensions of complexity variation in gender systems (Audring, 2014, 2017; Di Garbo, 2016). Both degree of inflectional synthesis on the verb and number of gender distinctions can be interpreted straightforwardly from the perspective of language complexity as the number of parts in a system. The two case studies are presented independently in sections 3.2 and 3.3.

### 3.1. Materials and methods: demographic data

In order to investigate whether there are general patterns in how language structure adapts to social structure, we focus on demographic data. We correlate the linguistic phenomena under study with two sociolinguistic variables, the number of native speakers and the proportion of non-native speakers in the community. In this section we discuss the structure of these demographic data and their problems. The data and sources are provided in the Supplementary Material.

When defining the sociolinguistic features we largely follow Lupyan and Dale (2010) and Bentz and Winter (2013). We define the number of L1 speakers as the current number of speakers and the data is largely taken from the 19th edition of the *Ethnologue* (Lewis et al., 2016), which lists the number of speakers for all currently spoken languages in the database. To better scale the number of native speakers in both small and large languages, we take the base-10 logarithm of the number of L1 speakers (cf. Lupyan and Dale, 2010). The *Ethnologue* lists the number of speakers for a particular country and separately in all countries and in some cases also the size of the ethnic population. The latter may be helpful and indicative of the relative size of the population before the number of speakers began to drastically decline as, for instance, in North America (e.g., Nichols, 2009). Here we use the number of speakers in all countries. One problem with the number of speakers is that changes in the speech community can sometimes be very quick, whereas changes in grammar are generally slower (cf. Sinnemäki, 2009). For this reason, it is unclear whether the current size of a speech community (or even the current size of the corresponding ethnic community) would reflect the situation at the time of writing the grammar or at the time in which the grammatical structures that are now captured in grammatical descriptions were developed. Numbers of native speakers should thus be conceived of as mere estimations, even when based on the most recent census.

The proportion of L2 speakers in the community is defined here as the proportion of non-native speakers in the whole speech community, where the size of the whole speech community includes both native and non-native speakers [that is, as *L*2/(*L1*+*L*2)] (Bentz and Winter, 2013). This measure is meant to estimate the likelihood that the grammar is affected by the presence of a particular proportion of population speaking the language as an L2. Some researchers have used a cut-off point for the proportion of non-native speakers. For instance, Kusters (2003, p. 41) defined his type 2 communities as those in which more than half of the speech community were adult L2 learners. On the other hand, a reviewer suggested that maybe there is some cut-off point after which the population size is large enough to act as a buffer against effects from the L2 population. While this is an interesting suggestion, there is some evidence actually to the contrary. McWhorter (2007) argues that especially the languages of large empires tend to be susceptible to simplifying effects from a large L2 population. Wray and Grace (2007) even suppose that bigger languages have more contact with surrounding languages. This latter point is not supported by our data, which instead suggests that there is some tendency for large languages to have lower proportions of L2 speakers, as indicated by the negative correlation (albeit not consistently significant) between log number of L1 speakers and the proportion of L2 speakers below. We return to this briefly in section 3.2.2. Overall, in the spirit of Bentz and Winter (2013), we hypothesize that the proportion of L2 speakers is best seen as a continuum, since there are no clear, theoretically motivated cut-off points between the two endpoints.

A reviewer also suggested that perhaps the raw number of L2 speakers would be a better predictor than the proportion of L2 speakers. Since the number of L1 speakers is used in counting the proportion of L2 speakers, this might increase collinearity owing to the mathematical interconnectedness between the number of L1 speakers and the proportion of L2 speakers. We do not think that using the number of L1 speakers in counting the proportion of L2 speakers is a problem to us. Log number of L1 speakers did not correlate significantly with the proportion of L2 speakers when semi-speakers were excluded (*r* = −0.147;*df* = 63;*p* = 0.24), only when they were included (*r* = −0.374;*df* = 71;*p* = 0.001) and it is the former measure that is our primary estimate for the proportion of L2 speakers (more on semi-speakers below).

There are also some problems related to the availability and reliability of the data that need to be addressed. While the data for the number of speakers are readily available in the *Ethnologue*, data on L2 speakers are available only for a small proportion of languages in the *Ethnologue*. Alternative sources are sporadic and poorly representative of the world's languages. In our sample this meant that we were able to obtain estimates for L2 data for roughly 70 languages.

The L2 data is problematic for two more reasons. One is that there is a range of speaker types that have been identified in the literature and not necessarily all sources use the same typology of speaker types. Grinevald (2003), for instance, divides speakers into 1. native speakers, 2. semi-speakers, 3. terminal speakers, and 4. rememberers. Native speakers are fluent, semi-speakers range from near-fluent to limited L2 speakers, terminal speakers are the last speakers of a dying language, and rememberers are speakers who have lost much of their earlier fluency in the language. In this classification most L2 speakers would be classified as semi-speakers. But it is not always clear what is counted as “L2 speaker.” Sources that focus more on language acquisition or database-building make a difference between native speakers and L2 speakers, but they do not necessarily distinguish L2 speakers from semi-speakers. Yet sometimes this distinction is made, as is done in the *Ethnologue*, which distinguishes L2 speakers from semi-speakers. The latter is possibly reserved as a characteristic speaker-type in situations of language endangerment in which the last fluent speakers are the elders of the community who do not accept the younger generation's error prone talk (cf. Thomason, 2015). But this is not quite clear from the *Ethnologue*, since the figures for L2 speakers are defined for all non-native speakers irrespective of their level of competence in the target language. These issues lead to possible problems in the comparability of the numbers reported in the sources. For the purpose of this paper we assume that the problems are not too great.

The second problem with the L2 data concerns the often poor quality of the data. The compilers of the *Ethnologue* are well aware of this and report in “Ethnologue Global Dataset” that they originally “refrained from including these data due to” problems with adequacy of the data^3^. However, they finally published the data because the customer demand was very high. Although the data is continually updated, estimating the number of L2 speakers is very difficult and involves a considerable amount of guesswork. For instance, the number of L2 speakers for Bengali, the main language of Bangladesh, was estimated to be at 140 million speakers in the 17th edition of the *Ethnologue*, published in 2014. This many L2 speakers constitute 56% of the whole speech community of Bengali in Bangladesh (including native and non-native speakers). However, the latest 20th edition of the *Ethnologue* (published in 2017) reports that there are 19.2 million L2 speakers of Bengali in Bangladesh, which is not more than 9.7% of the Bengali-speaking population in Bangladesh. In a similar way, the number of L2 speakers of Russian was about 30 million in the 2010 census (cf. the 19th edition of the *Ethnologue*), but according to Arefyev (2012) (via the 20th edition of the *Ethnologue*) the number of L2 speakers of Russian is closer to 113 million. Our point is not to criticize the data in the *Ethnologue*, because of all available language databases that contain information on speech community size this is still the largest and most reliable source. Rather, we argue that any database that aims to collect information on this type of figures would run into the same problems. When facing such degree of uncertainty with the data, one possibility is to average the reported figures (e.g., Bentz and Winter, 2013). We decided not to use averages but to take the data from the sources that were most recent or that we evaluated as the most reliable.

In order to explore whether the number of semi-speakers, as usually reported for small endangered languages, had an effect on the results, we conducted the statistical models by including the number of semi-speakers in the L2 data, but also report results about the models in which the L2 figures did not include the number of semi-speakers. The fact that semi-speakers have low competence of the target language may suggest that they may use simplified language with transfer effects from the native language. This kind of pidginization has been hypothesized to influence language structures in the target language. However, a high number of semi-speakers may not necessarily be indicative of the kind of sociolinguistic situation that has been hypothesized as having an influence on language structure. For instance, the situation of many North American Indian communities is such that the elders speak the language which the younger generation learns only as a L2. The elders may not accept the language of the younger generation, who may in turn feel inferior because of their bad knowledge of the language. This suggests that, in these and similar contexts, it is unlikely that the language use of the semi-speakers would simplify the language of the whole community.

### 3.2. Study 1: morphological complexity of the verb

#### 3.2.1. Materials and methods

The data for inflectional synthesis come from the *AUTOTYP* database, thus we follow its definition of the phenomenon. The database contains information on the degree of inflectional synthesis of verbs but not of other parts of speech. Here we provide succinct description of the definitions but guide the reader to Bickel and Nichols (2007, 2013) for further details (see also section 2.1). The material for inflectional synthesis is provided in the Supplementary Material.

According to Bickel and Nichols (2013) the degree of synthesis measures the number of morphological categories expressed per word in a maximally inflected verb form. The notion of maximally inflected word form refers to the fact that verbs can vary in terms of their synthesis within a language: the English past tense is marked with an affix *-ed* and the future tense with a separate word *will* so the past tense is more synthetic than the future tense in English. The data set codes the most synthetic verb forms in each sample language and registers the maximal number of categories per verb. For English this approach counts two categories, namely agreement (third person in present tense) and tense (past tense -*ed*). The counted categories do not have to coincide in the same verb form in language use, and often they do not.

Our hypothesis is that an inverse relationship exists between degree of inflectional synthesis on the verb and demographic factors. To assess this relationship we constructed generalized linear mixed effects models (GLMMs) using the package glmmADMB (Fournier et al., 2012; Skaug et al., 2016) in R (R Core Team, 2017). Mixed models have been recently applied and discussed in language typology by Sinnemäki (unpublished) and Jaeger et al. (2011). We used glmmADMB instead of the more popular lme4 package because the maximal models (see below) converged better with the former and because glmmADMB also offers ways of dealing with zero-inflated variables (see section 3.3.1)^4^. In addition, in models involving the number of L1 speakers the L1 population sizes were set to 50 when the actual number of L1 speakers was 50 or less. In doing so we follow Lupyan and Dale (2010). They do not explain why they manipulated the number of speakers in this way but the reason might be that for such small speech communities the numbers of speakers may be very unreliable.

**Figure 1:**
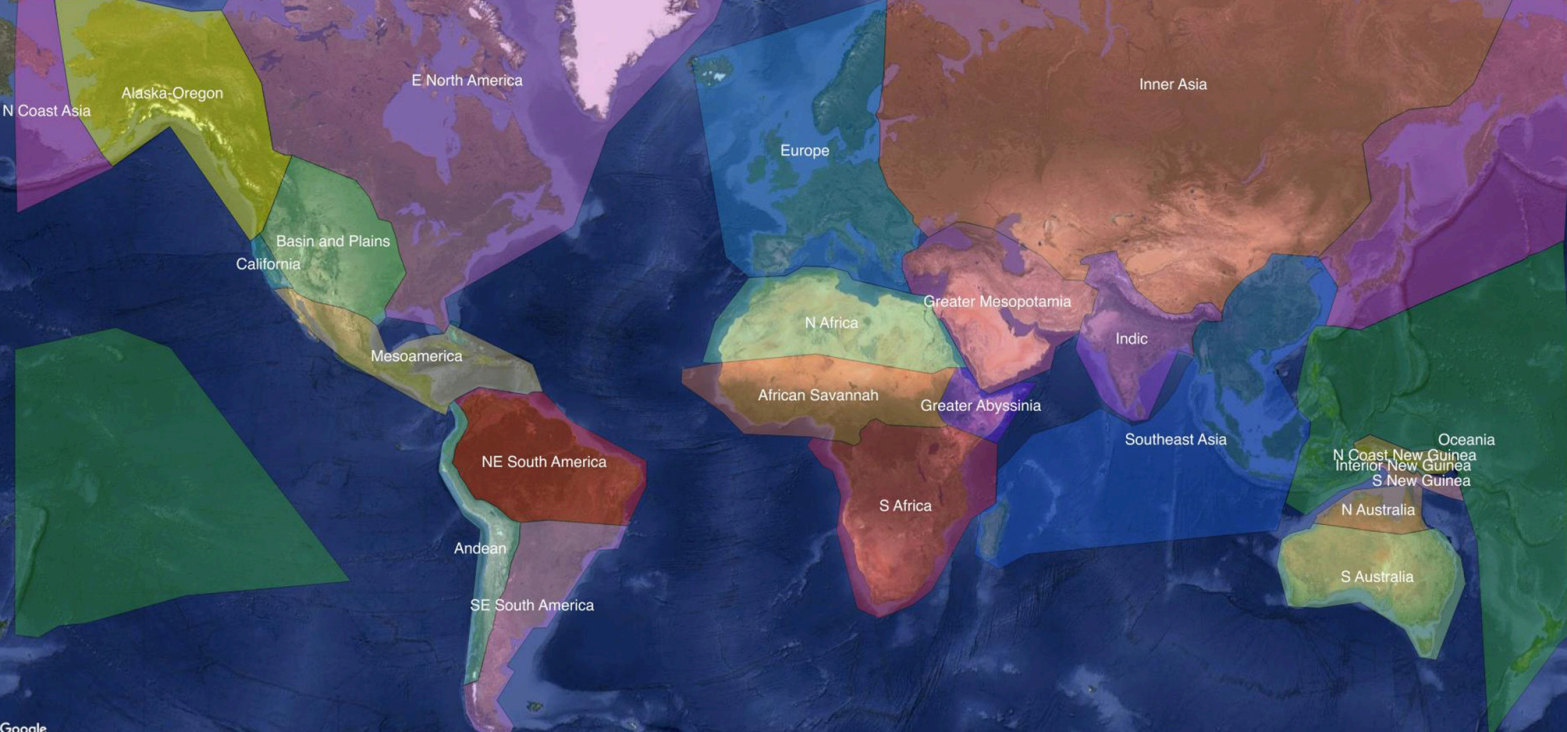
The 24 areas of the *AUTOTYP* on a world map (Bickel et al., 2017; used under CC-BY 4.0 license).

We constructed four models for this case study. The model designs are similar except for the predictors; the model names and their predictors are listed in Table 2. In all of the models the degree of inflectional synthesis was the response and the random structure was the same: *AUTOTYP* stocks were used as a grouping factor for genealogical affiliation and the 24 areas of *AUTOTYP* as the grouping factor for geographical areas. Stocks are the highest level in the genealogical taxonomy of *AUTOTYP*, roughly corresponding to language families in *WALS*. We prefer the *AUTOTYP* stocks to the *WALS* families because they are generally more conservative and do not posit problematic higher level families such as Altaic. The 24 areas of *AUTOTYP* consist of areas such as California, Europe, and Southeast Asia as well as 21 additional areas that are roughly parallel in size. Figure 1 illustrates these areas on a world map.

**Table 2:** Model names and predictors in case study 1.

**Model name**	**Predictor(s)**
SYNTHESIS.L1	log number of L1 speakers
SYNTHESIS.L2	proportion of L2 speakers (excluding semi-speakers)
SYNTHESIS.L2+	proportion of L2 speakers (including semi-speakers)
SYNTHESIS.ALL	log number of L1 speakers and proportion of L2 speakers (including semi-speakers)

Our models are maximal in that they include all the theoretically motivated random intercepts and slopes. In the light of recent debates, maximal models are preferred in mixed models since especially models without random slopes are susceptible to produce spurious results (Schielzeth and Forstmeier, 2009; Barr et al., 2013). However, models containing random slopes may lead to overfitting and the random effect variances being zero or approaching zero. To improve our models we tested whether some of the random slopes could be removed. For mixed models *p*-values can be derived by using maximum likelihood ratio tests, which can be applied for both fixed and random effects. To evaluate the *p*-values of effects we compared the likelihood ratio of a model with the variable of interest to that of a simpler model without the variable of interest (e.g., Baayen et al. 2008; Barr et al. 2013).

The degree of inflectional synthesis is discrete count data, ranging from 0 to 14, and therefore we used Poisson regression to model the data. Poisson distribution assumes that the sample mean is identical with the sample variance. The dispersion ratios in all the models were not significantly different from 1 (see Table 3), which means that the assumption of Poisson regression about identical sample mean and variance was met.

**Table 3:** Dispersion ratio and deviance from 1 for models in case study 1.

**Model name**	**Dispersion ratio**	**Estimation of deviance**
SYNTHESIS.L1	0.84	$\chi_{\left(311\right)}^{2}=261.5;p=0.98$
SYNTHESIS.L2	1.01	$\chi_{\left(57\right)}^{2}=57.5;p=0.46$
SYNTHESIS.L2+	0.85	$\chi_{\left(65\right)}^{2}=55.4;p=0.79$
SYNTHESIS.ALL	0.96	$\chi_{\left(64\right)}^{2}=61.3;p=0.57$

#### 3.2.2. Results

**Figure 2:**
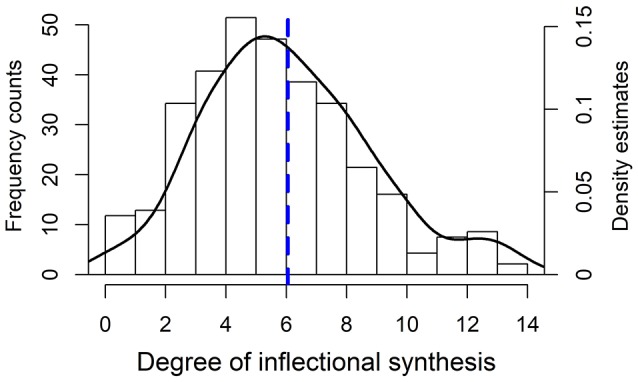
Frequency histogram and the superimposed density estimates for the degree of inflectional synthesis of the verb in the sample languages. The dotted vertical line represents the mean.

**Figure 3:**
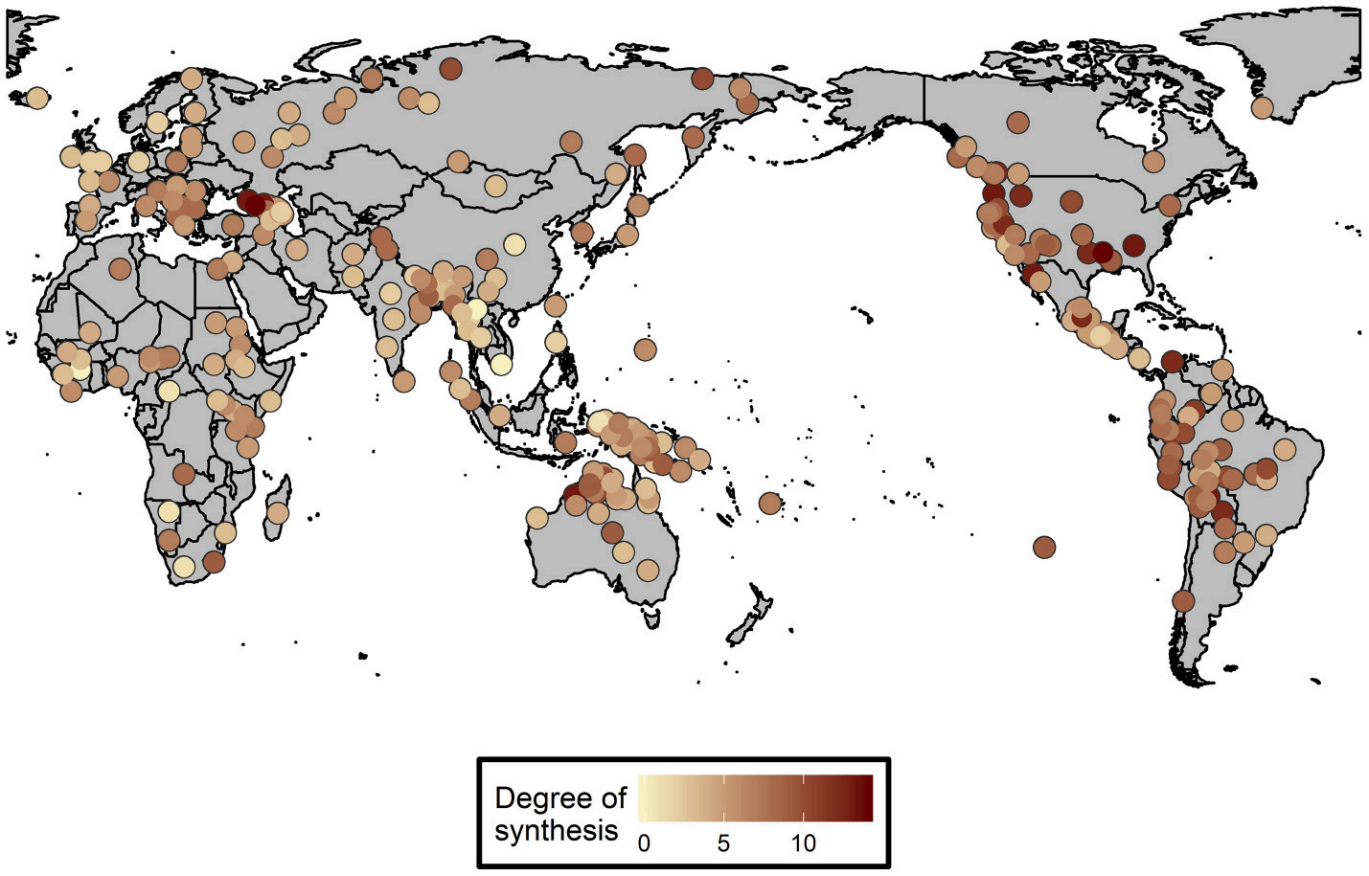
Degree of inflectional synthesis and its areal distribution on a world map.

The sample contains data on log number of native speakers and the degree of verbal inflectional synthesis in 309 languages. It was possible to get data on the proportion of L2 speakers in 65 languages and for an additional 8 languages on the number of semi-speakers. The histogram distribution of degree of inflectional synthesis is provided in Figure 2. The degree of inflectional synthesis is roughly normally distributed around a mean of six inflectional categories per verb. The areal distribution of the sample languages and their degree of inflectional synthesis is provided in Figure 3.

**Figure 4:**
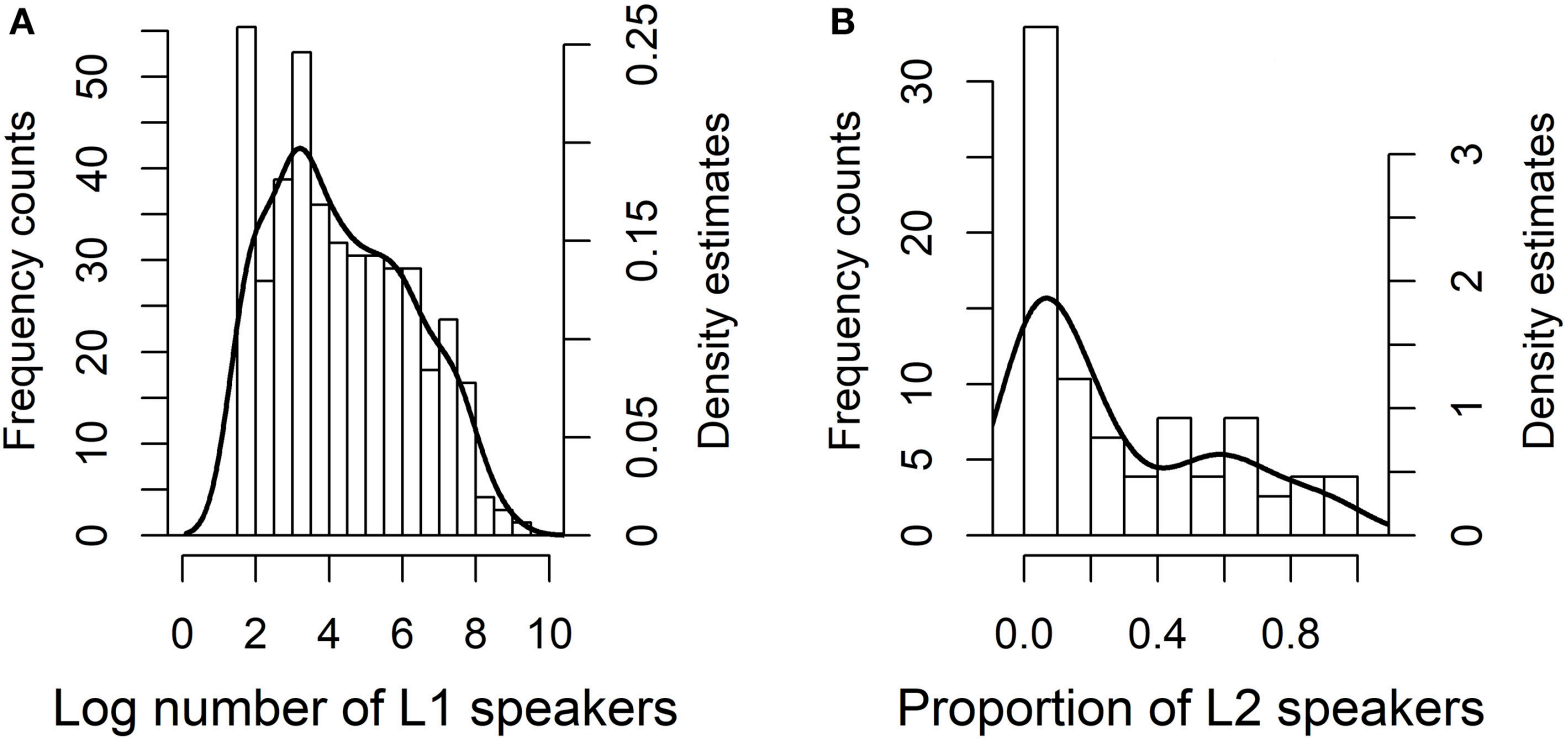
Frequency histograms and superimposed density estimates for the independent demographic variables in case study 1, for log number of native speakers on the left **(A)** and for the proportion of L2 speakers (including semi-speakers) on the right **(B)**.

The distribution of the demographic factors is shown in Figure 4. In the sample the median size of L1 populations was 14,100, which is much larger than the total median (7,000) for all spoken languages in the *Ethnologue*. This difference is possibly due to the fact that larger languages tend to be also better described than smaller languages. The median proportion of L2 speakers was 18% and that of semi-speakers 58%. The reason why the proportion of semi-speakers tends to be higher than that of L2 speakers is that the data for semi-speakers comes from small languages in North America with the kind of sociolinguistic situation we described in section 3.1.

**Figure 5:**
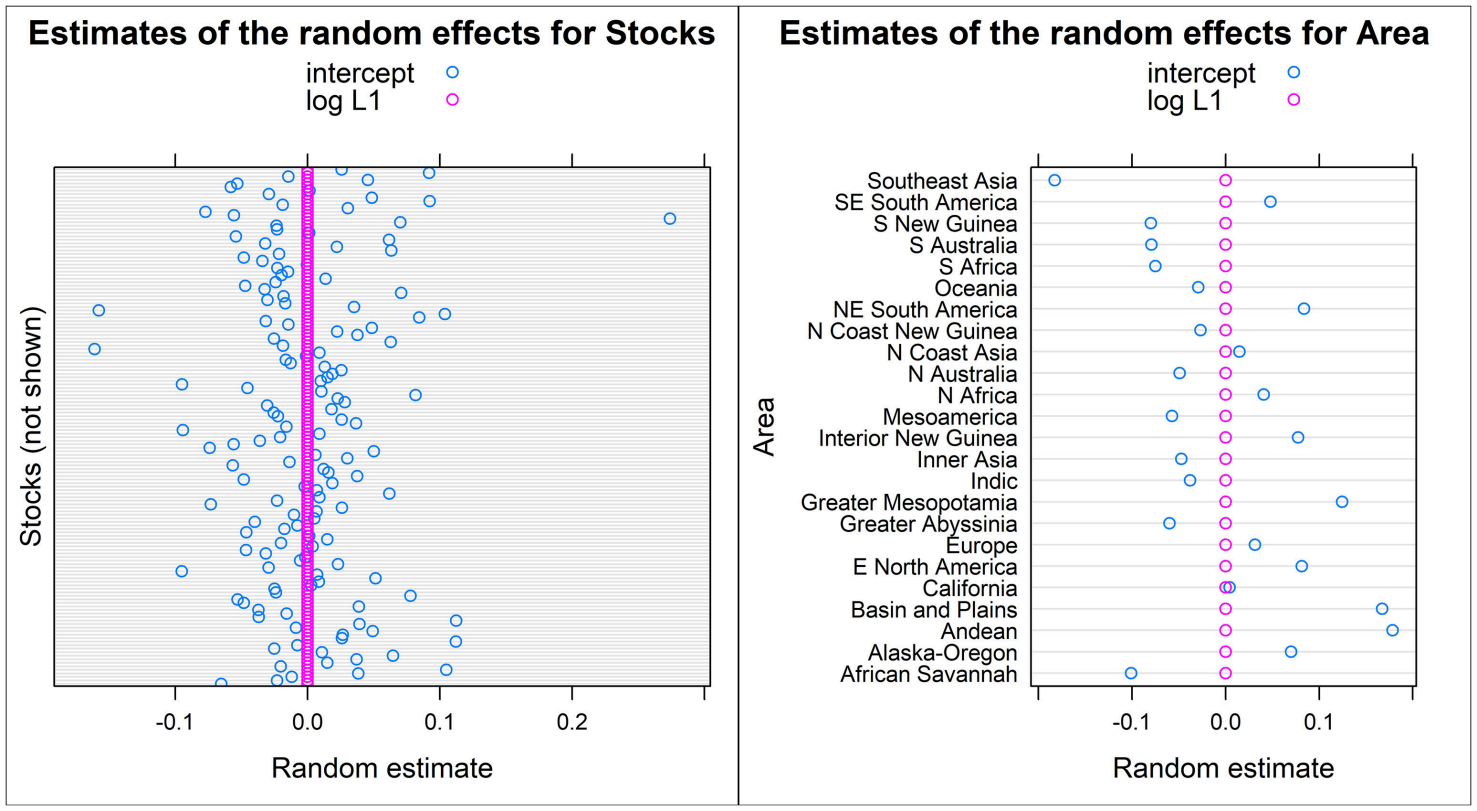
Random effect variation of model SYNTHESIS.L1. The left panel shows the estimates for the random intercept and random slope over Stocks and the panel on the right shows the estimates for the random intercept and random slope over Area.

According to the mixed logistic regression of the maximal model of SYNTHESIS.L1, log number of L1 speakers had a significant negative effect on the degree of inflectional synthesis [log(λ) = −0.077 ± 0.018; χ^2^(1) = 17.5;*p* = 0.000028]. However, while this maximal model converged the random effect variances for the slopes (both Stocks and Area) were very close to zero (see Figure 5), which suggests that the random slopes may be superfluous. The maximum likehood ratio tests confirm that both slopes may be removed from the model [random slope over Stocks: χ^2^(1) = 0.33;*p* = 0.57; random slope over Area: χ^2^(1) = 0.89;*p* = 0.35], which leaves us with a random intercept model.

According to the reduced model log number of L1 speakers had a significant negative effect on the degree of inflectional synthesis [log(λ) = −0.079 ± 0.018; χ^2^(1) = 17.9;*p* = 0.000023]. The negative coefficient and the significant p-value suggest that the hypothesis is confirmed. But because the estimate is rather small, the size of the speech community seems to have only a small impact on the degree of inflectional synthesis. Because in Poisson regression it is the log of the expected counts that is modeled, the coefficients can be transformed via inverse logarithm to better understand them. The coefficient for log of L1 speakers was −0.077 and its inverse logarithm is 0.926. This means that as the population size becomes 10 times larger (we used log10) the language will have on average 7.4% fewer inflectional categories per verb conditioned by the random effect structure.

To further assess the models' goodness of fit, we used Akaike Information Content (AIC) or its small sample equivalent AICc which is corrected for bias (Burnham and Anderson, 2002). AIC can be used to evaluate the importance of a predictor by considering to what extent adding the fixed effect reduces AIC. Lower values of AIC improve the model's fit and, therefore, the larger the reduction in AIC is, the more important the predictor (e.g., Baayen, 2013). As a rough guideline, if the difference in AIC between the models is <2, the models fit the data roughly equally well, that is, there is no significant difference between the models; if the difference is between 4 and 7 there is much less support for the model with the higher AIC value, that is, the AIC difference can be considered important; if the difference is 10 or greater, there is basically no support for the model with the higher AIC value (Burnham and Anderson, 2002, p. 70–71). We compared the AIC values in the model which contained the log number of L1 speakers (*AIC* = 1459.9) to a model that contained only the random intercepts (*AIC* = 1475.8). Adding the fixed effect reduced the AIC by 15.9. Since the difference is >10, there is substantial support for the model that included the log number of L1 speakers. In other words, although the effect of the log number of L1 speakers on the degree of synthesis was small, it was nevertheless reasonable, as it clearly improved model fit^5^.

According to the mixed logistic regression of model SYNTHESIS.L2, the proportion of L2 speakers had an inverse effect on the degree of inflectional synthesis but this effect was not significant (log(λ) = −0.39 ± 0.23; χ^2^(1) = 3.04;*p* = 0.081). Again, while the maximal model converged the random effect variances for both Stocks and Area were very close to zero (of the magnitude of 1e-7), which suggests that some of the random structure may be superfluous. The maximum likelihood ratio tests confirm that both slopes may be removed from the model (random slope over Stocks: χ^2^(1) = 0.24;*p* = 0.63; random slope over Area: χ^2^(1) = 0.002;*p* = 0.96).

According to the reduced model the proportion of L2 speakers had an inverse and borderline significant effect on the degree of inflectional synthesis (log(λ) = −0.398 ± 0.21; χ^2^(1) = 3.798;*p* = 0.051). However, the borderline significant p-value makes the result somewhat uncertain. We further compared the AIC values in the model which contained the proportion of L2 speakers (304.2) to a model that contained only the random intercepts (306.0). Adding the fixed effect decreased the AIC only by 1.8, which provides further evidence that the proportion of L2 speakers has a negligible effect on the degree of inflectional synthesis.

**Figure 6:**
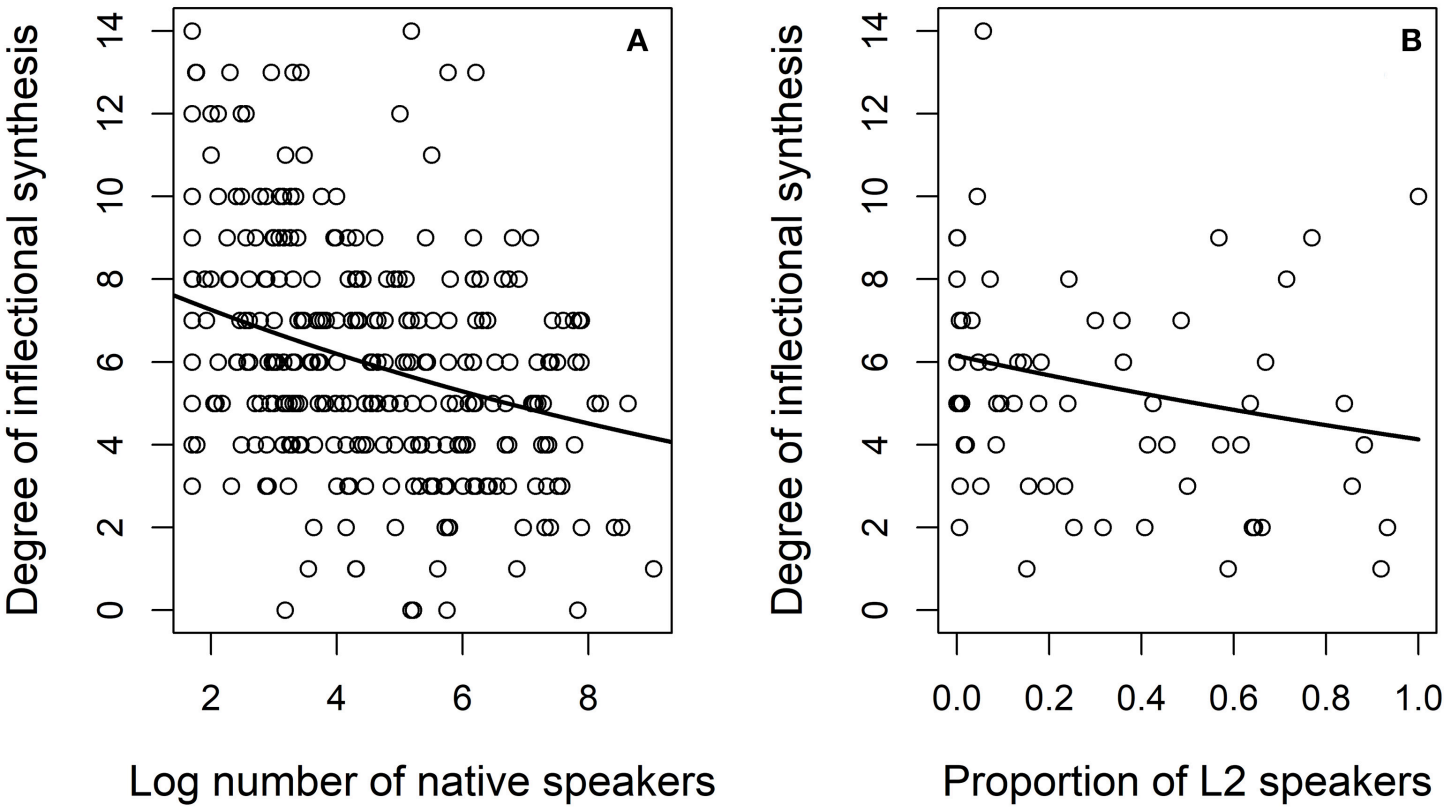
Degree of inflectional synthesis as a function of log number of speakers on the left **(A)** and for the proportion of L2 speakers on the right **(B)**.

Figure 6 presents the degree of inflectional synthesis as a function of the demographic variables in models SYNTHESIS.L1 and SYNTHESIS.L2. The curve indicates the fit of the mixed regression model. The figure on the left (Figure 6A) presents the fit to log number of L1 speakers. In communities with about 1,000 speakers or less [*log*_(1, 000)_ = 3] the predicted degree of synthesis is about 7 while it drops to about 5 in communities with a million or more L1 speakers [*log*_(1, 000, 000)_ = 6]. The downward slope is clear but not impressively large. The figure on the right (Figure 6B) presents the fit to the proportion of L2 speakers. There is a small downward trend so that in communities with few L2 speakers the predicted degree of synthesis is around 6, whereas in communities with close to 100% L2 speakers the predicted degree is about 4.

**Figure 7:**
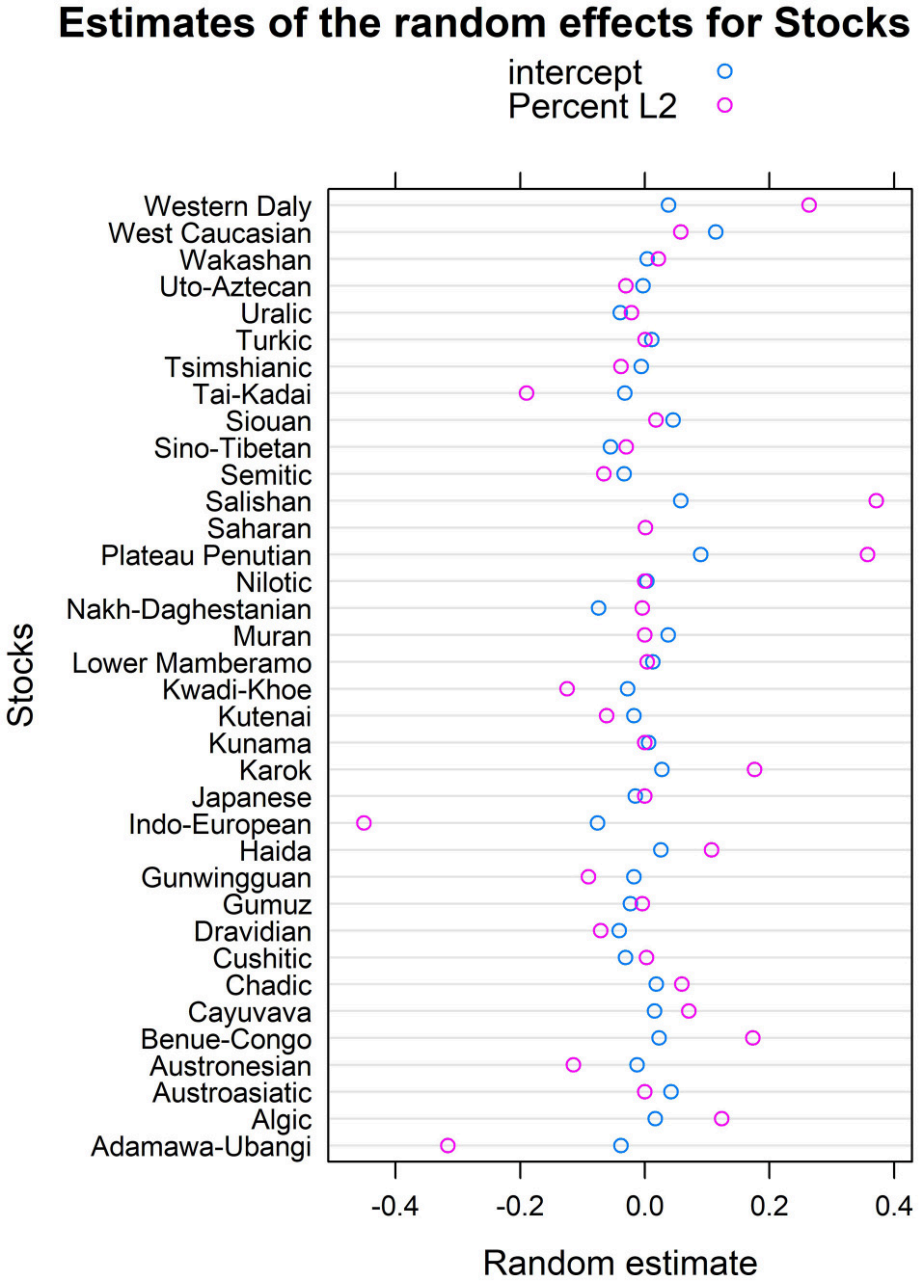
Estimates of the random effects for Stock in the reduced model SYNTHESIS.L2+.

According to the mixed logistic regression of model SYNTHESIS.L2+, the proportion of L2 speakers (including semi-speakers) had an inverse effect on the degree of inflectional synthesis but this effect was not significant [log(λ) = −0.27 ± 0.24; χ^2^(1) = 1.32;*p* = 0.25]. We again tested the random effect structure with maximum likelihood ratio tests and removed the random slope for Area but not that for Stocks [random slope over Stocks: χ^2^(1) = 3.96;*p* = 0.047; random slope over Area: χ^2^(1) = 1.83;*p* = 0.18]. According to the reduced model the proportion of L2 speakers (including semi-speakers) had an inverse but non-significant effect on the degree of inflectional synthesis [log(λ) = −0.23 ± 0.23; χ^2^(1) = 1.07;*p* = 0.30]. The negative coefficient provides support for the hypothesis but the non-significant *p*-value goes against the hypothesis. According to this model, the effect of L2 proportion on inflectional synthesis is largely lineage-specific. This is suggested by the significant random slope for Stock and by the large positive (e.g., in Salishan) and negative (e.g., in Indo-European) random variances for Stock (see Figure 7).

All in all when the effect of the demographic variables was researched in isolation only the number of L1 speakers had a clearly significant and negative effect on the degree of inflectional synthesis. The significant effect of the number of L1 speakers replicates the result by Lupyan and Dale (2010). However, compared to the proportion of L2 speakers the number of L1 speakers is a less direct measure of the kind of language contact effects that have been hypothesized to influence language structures (see section 2.3). For this reason it is somewhat surprising that it was the less direct measure of language contact effects that had a significant effect on language structures in the modeling. It is possible that this is mostly due to sample size. In the model SYNTHESIS.L1 the sample size was 309 languages but in the model SYNTHESIS.L2 the sample size was 65 languages. In order to test whether this result depended on sample size, we modeled the effect of the two demographic variables in the same model.

In model SYNTHESIS.ALL we model the effects of the log number of L1 speakers and the proportion of L2 speakers (excluding semi-speakers) in competition with one another. According to the mixed logistic regression of the maximal model, log number of L1 speakers had a significant inverse effect on the degree of inflectional synthesis [log(λ) = −0.12 ± 0.026; χ^2^(1) = 15.2;*p* = 0.000095]. The proportion of L2 speakers (excluding semi-speakers) had also an inverse effect and this time also a significant effect on the degree of inflectional synthesis [log(λ) = −0.47 ± 0.20; χ^2^(1) = 5.8;*p* = 0.016]. We again tested the random effect structure with maximum likelihood ratio tests because most of the random effect variances for the slopes (both Stocks and Area) were very close to zero (of the magnitude of 1e-7) and ended up removing all the random slopes (all were non-significant).

According to this reduced model the log number of L1 speakers had a significant inverse effect on the degree of inflectional synthesis [log(λ) = −0.10 ± 0.026; χ^2^(1) = 12.3;*p* = 0.00046] and so did the proportion of L2 speakers [log(λ) = −0.47 ± 0.19; χ^2^(1) = 6.58;*p* = 0.010]. For the purpose of model comparison, we modeled the log number of L1 speakers in isolation from the proportion of L2 speakers but just for this smaller data set (*n* = 65), keeping the random structure identical (that is, modeling just the random intercepts). In this model the log number of L1 speakers again had a significant but slightly smaller inverse effect on the degree of inflectional synthesis [log(λ) = −0.09 ± 0.025; χ^2^(1) = 9.5;*p* = 0.0021] than when modeling the log number of L1 speakers in the same model with the proportion of L2 speakers. The coefficient for log of L1 speakers was −0.10 and its inverse logarithm is 0.905. This means that (in this smaller sample) as the population size becomes 10 times larger the language will have on average 9.5% fewer inflectional categories per verb conditioned by the random effect structure. The coefficient for the proportion of L2 speakers was −0.47 and its inverse logarithm is 0.625. This means that languages spoken by communities with 100% L2 speakers have about 37.5% fewer inflectional categories per verb than those with no L2 speakers conditioned by the random effect structure.

**Figure 8:**
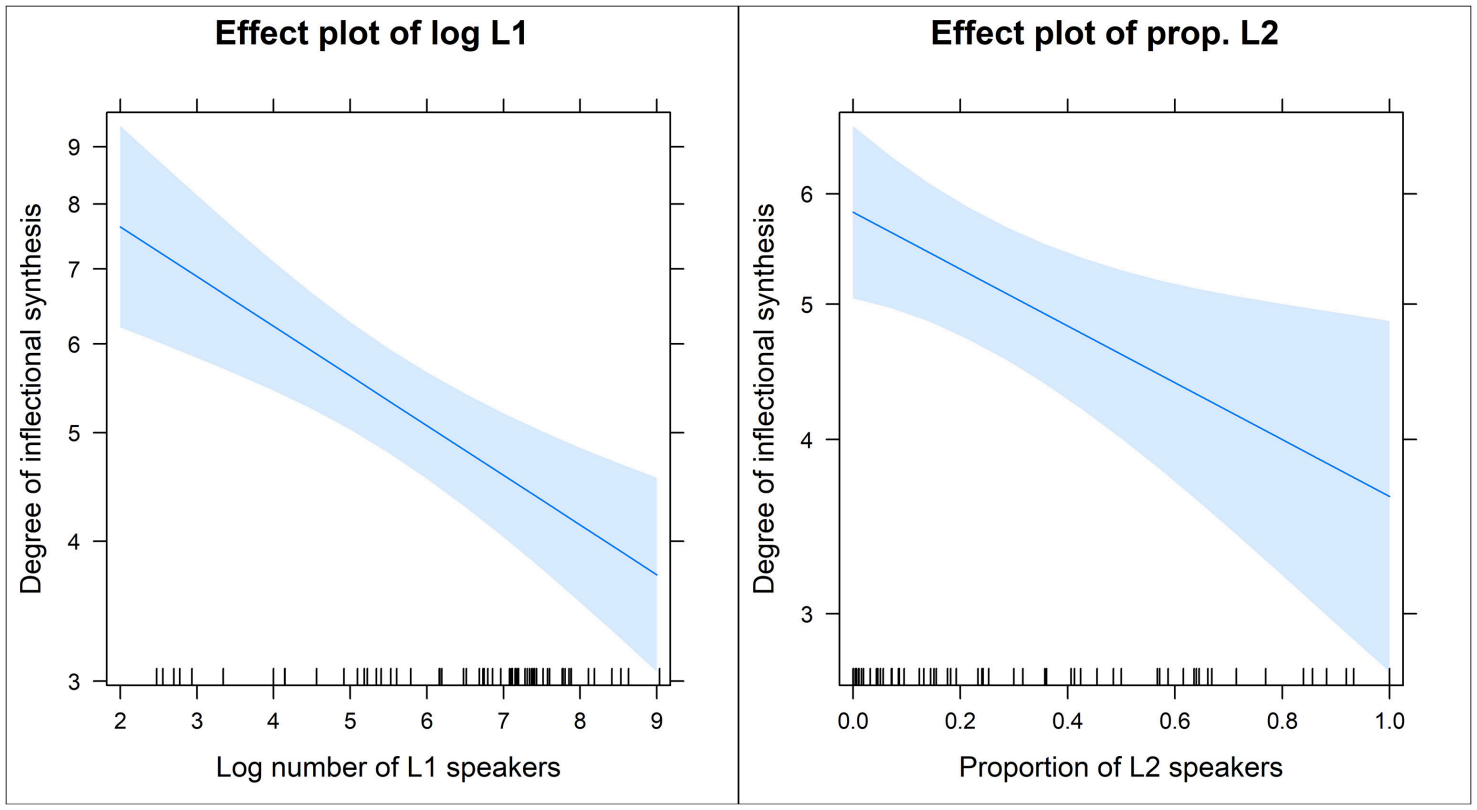
Effect plots of the model predictors in model SYNTHESIS.ALL (log number of L1 speakers on the left and the proportion of L2 speakers on the right).

Figure 8 presents the effect plots for the model predictors in model SYNTHESIS.ALL^6^. The plots present the predictors' values on the x-axis and the predicted values of the response on the y-axis. Based on the effect plot for log L1 speakers as the predictor, the predicted degree of synthesis drops from roughly eight categories in communities with about 10 speakers [*log*_(100)_ = 2] to about four in communities with 100 million or more L1 speakers [*log*_(100, 000, 000)_ = 8]. The downward slope is very clear. Based on the effect plot for the proportion for L2 speakers, the predicted degree of synthesis drops from roughly six categories in communities with no L2 speakers to about four in communities with about 80% or more L2 speakers. There is a downward slope but not as steep as for the log number of L1 speakers.

For model comparison we used AICc; the results are reported in Table 4 in decreasing order of AICc. Based on the AICc values the model (1) which contained only the random intercepts but no fixed effects had the largest AICc value (306.4) and, therefore, it is the worst of the four models. In model (2) the proportion of L2 speakers was added as a fixed effect to the random intercepts-model and this decreased the AICc by 1.5 compared to model (1). This decrease is small and suggests that modeling the proportion of L2 speakers in isolation from the log number of native speakers produces a negligible effect. In model (3) the log number of L1 speakers was added to the random intercepts-model and this decreased the AICc by 7.2 compared to model (1). This large reduction suggests that the log number of L1 speakers has a reasonable effect on the degree of inflectional synthesis. In model (4) the proportion of L2 speakers was added as a fixed effect to model (3), which gives us the full model that contained the random intercepts and both of our fixed effects. In the full model the AICc value was the smallest, being 4.2 smaller than in model (3). We further used Akaike weights (the right-most column in Table 4) to compare these four models to one another (Burnham and Anderson, 2002). The Akaike weights scale the differences in the models' AIC values to a scale of 1 and thus provide an easy and effective way to interpret the models' AIC differences^7^. Based on the Akaike weights, the model (4) which includes both the log number of L1 speakers and the proportion of L2 speakers has 88.4% chance of being the best model among our four models. These results suggests that modeling both demographic factors in the same model significantly improves the model fit compared to modeling them in isolation from one another^8^.

**Table 4:** Results of model comparison for the reduced model SYNTHESIS.ALL. The full model includes both the log number of L1 speakers and the proportion of L2 speakers.

**Model**	**Model structure**	**Df**	**AICc**	**Reduction in AICc**	**Akaike weights**
(1)	Only random structure	3	306.4	0.0	0.003
(2)	Proportion of L2	4	304.9	1.5	0.006
(3)	Log number of L1 speakers	4	299.2	7.2	0.107
(4)	Full model	5	295.0	4.2	0.884

Thus, to summarize, the log number of L1 speakers has a significant effect on the degree of inflectional synthesis both in the larger sample (SYNTHESIS.L1; *n* = 309) and in the smaller sample (SYNTHESIS.ALL; *n* = 65). Conversely, the proportion of L2 speakers has a clearly significant effect on the degree of inflectional synthesis only when modeling it in competition with the number of L1 speakers (*p* = 0.010) but not when modeling it in isolation (*p* = 0.051). These results are confirmed by comparing the AIC values.

#### 3.2.3. Discussion

Two of the four statistical tests that we carried out to investigate the effect of population data on the degree of inflectional synthesis yielded significant results. Altogether these findings replicate and expand on previous research (Lupyan and Dale, 2010; Bentz and Winter, 2013) and suggest that the hypothesis whereby verbal inflectional synthesis adapts to demographic variables is corroborated by the present data set.

Our first model (SYNTHESIS.L1) replicated the earlier findings by Lupyan and Dale (2010). However, our results were based on a data set (309 languages) that was more than two times larger than the data set (145 languages) in Lupyan and Dale (2010). We also used the original exact counts for the degree of inflectional synthesis from *AUTOTYP* rather than the conflated count categories from the *WALS*.

We then estimated the proportion of L2 speakers in the whole speech community in the spirit of Bentz and Winter (2013). In their study the proportion of L2 speakers had a significant inverse effect on the number of case distinctions but, importantly, the size of the speech community did not. The fact that in our models the proportion of L2 speakers (whether including or excluding semi-speakers) did not have a clearly significant effect on the degree of verbal inflectional synthesis suggests that the proportion of L2 speakers alone is not a sufficient predictor of adaptive effects for all kinds of different linguistic structures, although it may be sufficient for some, such as number of cases. This result is in line with the hypotheses of Trudgill (2011b), who argues that single sociolinguistic features may not be sufficient for showing correlations between language structure and sociolinguistic structure and that richer models of the sociolinguistic environment are necessary instead.

We also contrasted two measures for the proportion of L2 speakers, namely, one including semi-speakers and the other excluding them. While in the latter model (SYNTHESIS.L2) the proportion of L2 speakers was borderline significant, in the former (SYNTHESIS.L2+) it was not. In addition, in the former model the slope for Stocks was significant. This result may be related to the observation in section 3.2.2 that the median proportion of L2 speakers was much smaller than the median proportion of semi-speakers, all of which came from small languages of North America. In other words, the large median proportion of semi-speakers suggests a different sociolinguistic environment, and thus different conditioning factors for those languages for which the number of semi-speakers was reported compared to those for which the number of L2 speakers was reported. For future research it may thus be necessary to treat L2 speakers separately from semi-speakers, to the extent that this is analytically possible.

Lastly in our model SYNTHESIS.ALL we included both the log number of L1 speakers and the proportion of L2 speakers (excluding semi-speakers) in the same model, which produced a set of interesting results. First, the number of L1 speakers had a significant effect even with the smaller sample (compared to model SYNTHESIS.L1). This result suggests that the number of L1 speakers is an important predictor of the degree of verbal inflectional synthesis and that the result in model SYNTHESIS.L1 was not just a consequence of larger sample size. Most interestingly, both our sociolinguistic factors had a significant inverse effect on the degree of inflectional synthesis when modeled as fixed effects in the same model and this model was also the best among competing models when using Akaike weights. In contrast, the proportion of L2 speakers did not have a clearly significant effect on the degree of inflectional synthesis when modeled in isolation (model SYNTHESIS.L2 and model SYNTHESIS.ALL). These results are in line with Trudgill (2011a,b)'s predictions. According to Trudgill, the sociolinguistic environment that attracts adaptation in the complexity of language structures cannot be systematically characterized by single sociolinguistic features, such as population size, but demands richer data. He further suggests that three sociolinguistic factors are decisive, namely, population size (here roughly the number of L1 speakers), degree of language contact (that we approximate by measuring the proportion of L2 speakers in the speech community), and the density of social networks. While our models did not include a factor for density of social networks, they still provided improved results compared to modeling the sociolinguistic factors in isolation. For future research our results suggest that the kind of sociolinguistic environment that may attract changes in the complexity of language structures cannot be easily captured by single demographic factors, but should preferably include information about population size, degree of contact vs. isolation, and possibly also other factors.

### 3.3. Study 2: morphological complexity and grammatical gender

#### 3.3.1. Materials and methods

We collected data on the number of genders in 345 languages. The material is provided in the Supplementary Material. The data is largely based on Sinnemäki (unpublish) and Corbett (2013a) and therefore we follow the definitions in these two studies.

As outlined in section 2.2, we define gender as a grammatical strategy that groups nouns into classes. These classificatory distinctions are not necessarily marked on nouns, but must be marked on clausal constituents that are in a syntactic relationship (also known as agreement) with nouns.

The number of genders in a language was counted based on number of distinguishable agreement classes. Usually a gender class is marked consistently across inflectional paradigms. However, often not all distinctions are present in all paradigms, as is the case in Mufian (Table 1). For instance, verb prefixes in Mufian are identical in classes 1, 2, and 3 in the plural, but in the singular the classes are distinguished from one another. For this reason each of these classes was counted as a separate gender in Mufian; all sample languages were analyzed with the same principles.

Our hypothesis is that an inverse relationship exists between the number of genders and the demographic factors used as independent variables. Similarly to case study 1, we constructed generalized linear mixed effects models using the package glmmADMB (Fournier et al., 2012; Skaug et al., 2016) in R (R Core Team, 2017) to assess the relationship between the number of genders and the demographic factors. The Poisson regression modeling is complicated by the large number of zeroes. The sample contains 345 languages but 200 (58%) of them have no genders. We accounted for this high number of zeroes by using zero inflation models offerred by glmmADMB. As in study 1, in this case study, too, we set the L1 population sizes to 50 when the actual number of L1 speakers was 50 or less (and for the same reasons; see section 3.2.1).

We constructed four models in this case study following the same principles as in case study 1 (see section 3.2.1). The model names and their predictors are listed in Table 5. In all of the models the number of genders was the response and the random structure was the same: *AUTOTYP* stocks were used as a grouping factor for genealogical affiliation and the 24 areas of *AUTOTYP* as the grouping factor for areas. However, models containing random slopes may lead to overfitting and the random effect variances being zero or approaching zero. To improve our models we tested whether some of the random slopes could be removed.

**Table 5:** Model names and predictors in case study 2.

**Model name**	**Predictor(s)**
GENDER.L1	log number of L1 speakers
GENDER.L2	proportion of L2 speakers (excluding semi-speakers)
GENDER.L2+	proportion of L2 speakers (including semi-speakers)
GENDER.ALL	log number of L1 speakers and proportion of L2 speakers (including semi-speakers)

The number of genders is discrete count data, ranging from 0 to 17, and therefore we used Poisson regression to model the data. Poisson distribution assumes that the sample mean is identical with the sample variance. However, the dispersion ratios met the assumption about identical sample mean and variance (that is, the dispersion ratios were not significantly different from 1) only in model GENDER.L1. In models GENDER.L2, GENDER.L2+, and GENDER.ALL the dispersion ratio was significantly different from 1 which means that the assumption about identical sample mean and variance was not met for these models (see Table 6). Our solution was to use negative binomial models for these three models and Poisson regression for GENDER.L1.

**Table 6:** Dispersion ratio and deviance from 1 for models in case study 2.

**Model name**	**Dispersion ratio**	**Estimation of deviance**
GENDER.L1	0.90	$\chi_{\left(337\right)}^{2}=301.6;p=0.92$
GENDER.L2	1.32	$\chi_{\left(57\right)}^{2}=75.5;p=0.051$
GENDER.L2+	1.35	$\chi_{\left(64\right)}^{2}=86.3;p=0.033$
GENDER.ALL	1.78	$\chi_{\left(57\right)}^{2}=101.2;p=0.00028$

#### 3.3.2. Results

**Figure 9:**
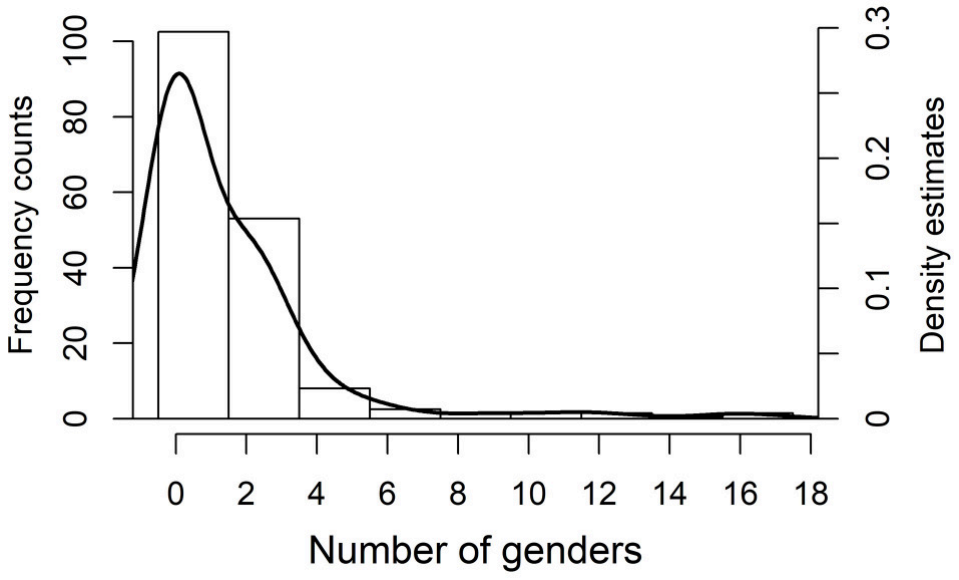
Frequency histogram and the superimposed density estimates for the number of genders in the sample languages (because no language has exactly one gender we smoothed over this absence by using biased cross-validation for bandwidth in density estimation).

**Figure 10:**
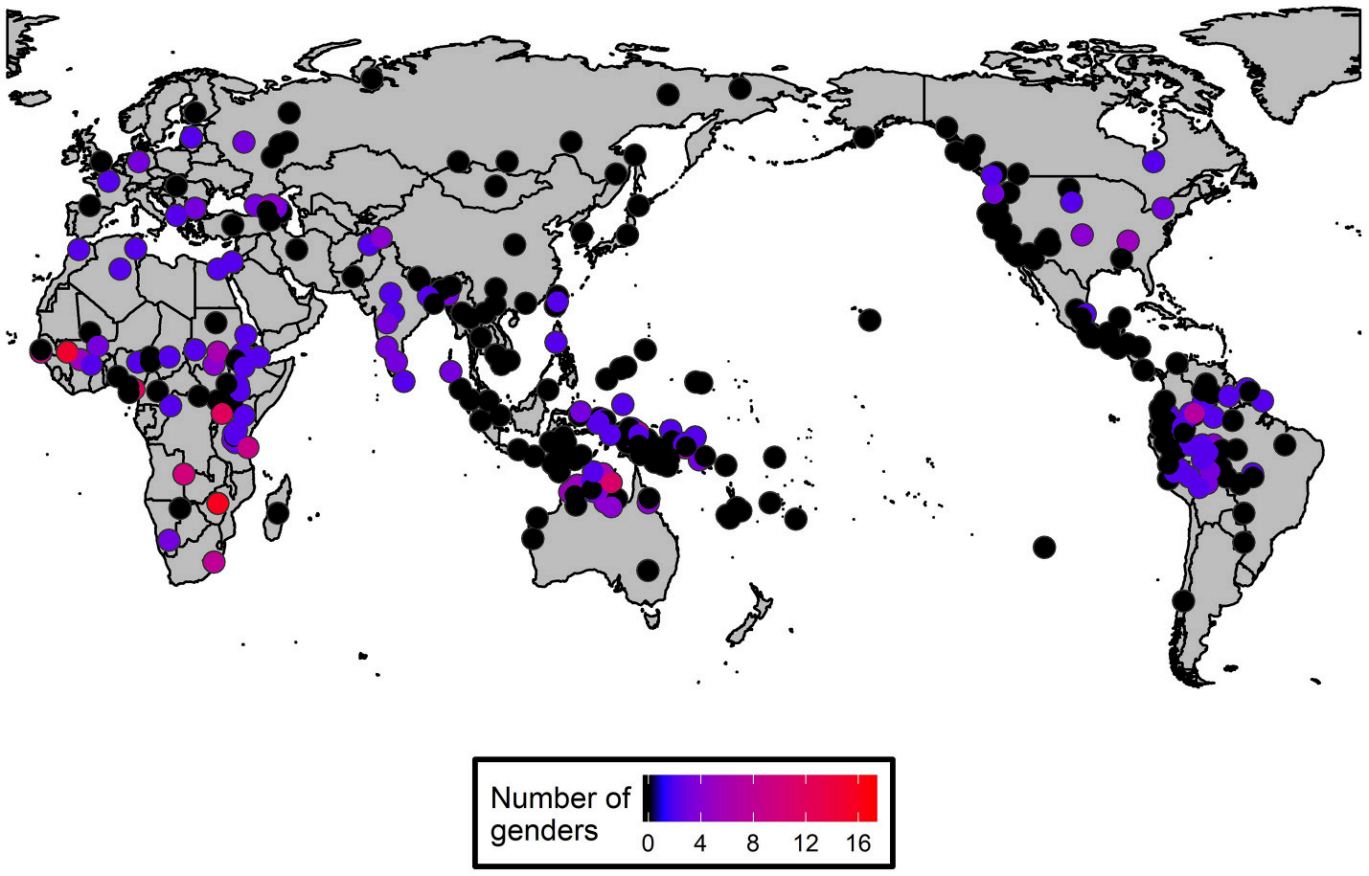
The distribution of number of genders on a world map (In the figure black dots represent languages with no gender and blue dots represent those with two genders. The deeper the red color, the more genders the language has).

The sample contains data on log number of native speakers and the number of genders in 345 languages. It was possible to get data on the proportion of L2 speakers in 65 languages and for an additional 7 languages on the number of semi-speakers. The distribution of the number of genders is shown in Figure 9. The number of genders has a roughly negative exponential distribution, that is, it is strongly skewed to the right. This kind of distribution is typical for typological variables (Cysouw, 2010). The areal distribution of number of gender is provided in Figure 10 on a world map.

**Figure 11:**
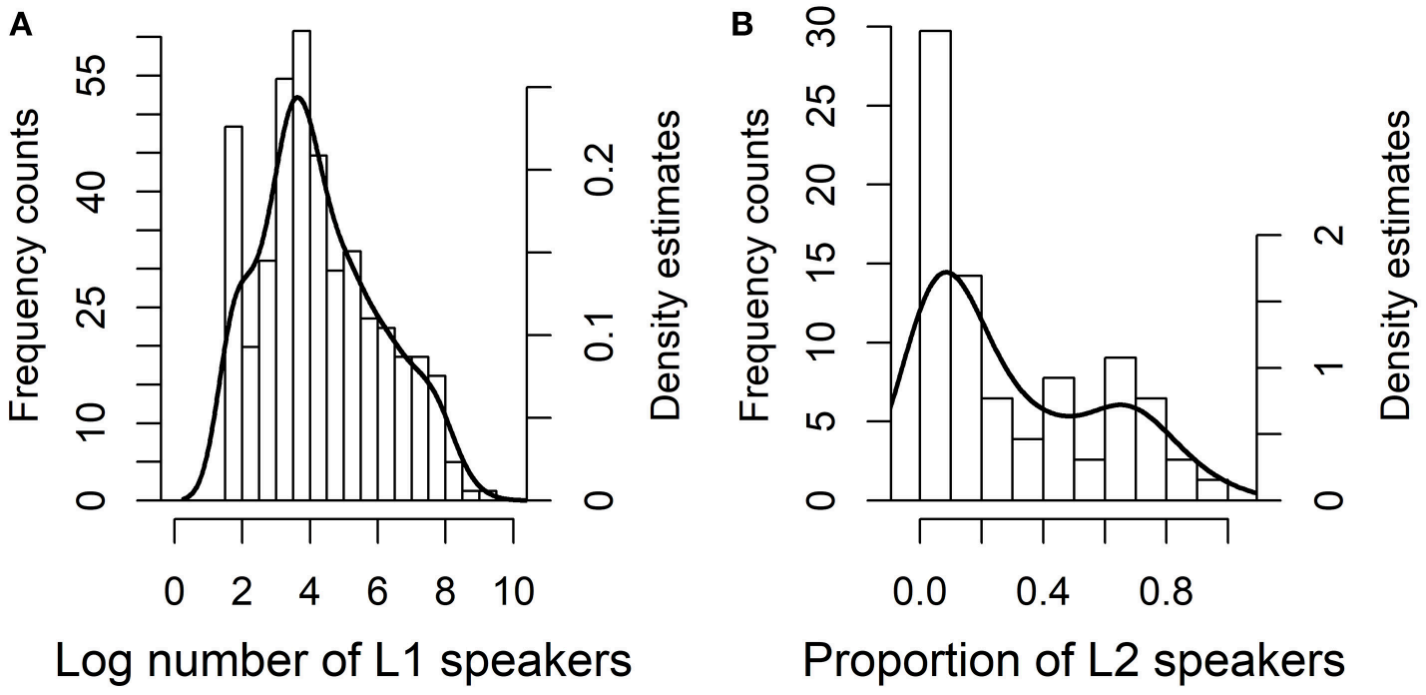
Frequency histograms and superimposed density estimates for the independent demographic variables in case study 2, for log number of native speakers on the left **(A)** and for the proportion of L2 speakers (including semi-speakers) on the right **(B)**.

The distribution of the demographic factors for the sample languages is shown in Figure 11. In this sample the median size of L1 populations was 10,000, which is somewhat smaller than in case study 1 but still larger than the total median of 7,000 for all spoken languages in the *Ethnologue*. The median proportion of L2 speakers was 19% and that of semi-speakers 58%. These figures are practically identical to those in case study 1 because roughly the same data was used.

According to the zero-inflated mixed logistic regression of the maximal model of GENDER.L1, log number of L1 speakers had a non-significant (positive) effect on the number of genders [log(λ) = 0.015 ± 0.069; χ^2^(1) = 0.048;*p* = 0.83]. However, while this maximal model converged the random effect variances for the slopes (both Stocks and Area) were very close to zero (of the magnitude of 1e-7). The maximum likehood ratio tests confirm that both slopes may be removed from the model [random slope over Stocks: χ^2^(1) = 0.14;*p* = 0.71; random slope over Area: χ^2^(1) = 1.49;*p* = 0.22]. According to the reduced model, the effect of log number of L1 speakers on the number of genders was non-significant [log(λ) = 0.024 ± 0.059; χ^2^(1) = 0.17;*p* = 0.68]. The non-significant p-value provides evidence that log number of native speakers has no effect on the number of genders.

According to the zero-inflated negative binomial mixed logistic regression of model GENDER.L2, the effect of the proportion of L2 speakers on the number of genders was negative but non-significant [log(λ) = −0.69 ± 0.49; χ^2^(1) = 1.1;*p* = 0.30]. Again, while the maximal model converged the random effect variances for both Stocks and Area were very close to zero (of the magnitude of 1e-7) and as a result the random slopes for both Area and Stocks were removed [random slope over Stocks: χ^2^(1) = 1.1;*p* = 0.29; random slope over Area: χ^2^(1) = 0.006;*p* = 0.94]. According to the reduced model the proportion of L2 speakers had an inverse and non-significant effect on the number of genders [log(λ) = −0.53 ± 0.61; χ^2^(1) = 0.59;*p* = 0.44]. Based on these results the proportion of L2 speakers has no effect on the number of genders.

**Figure 12:**
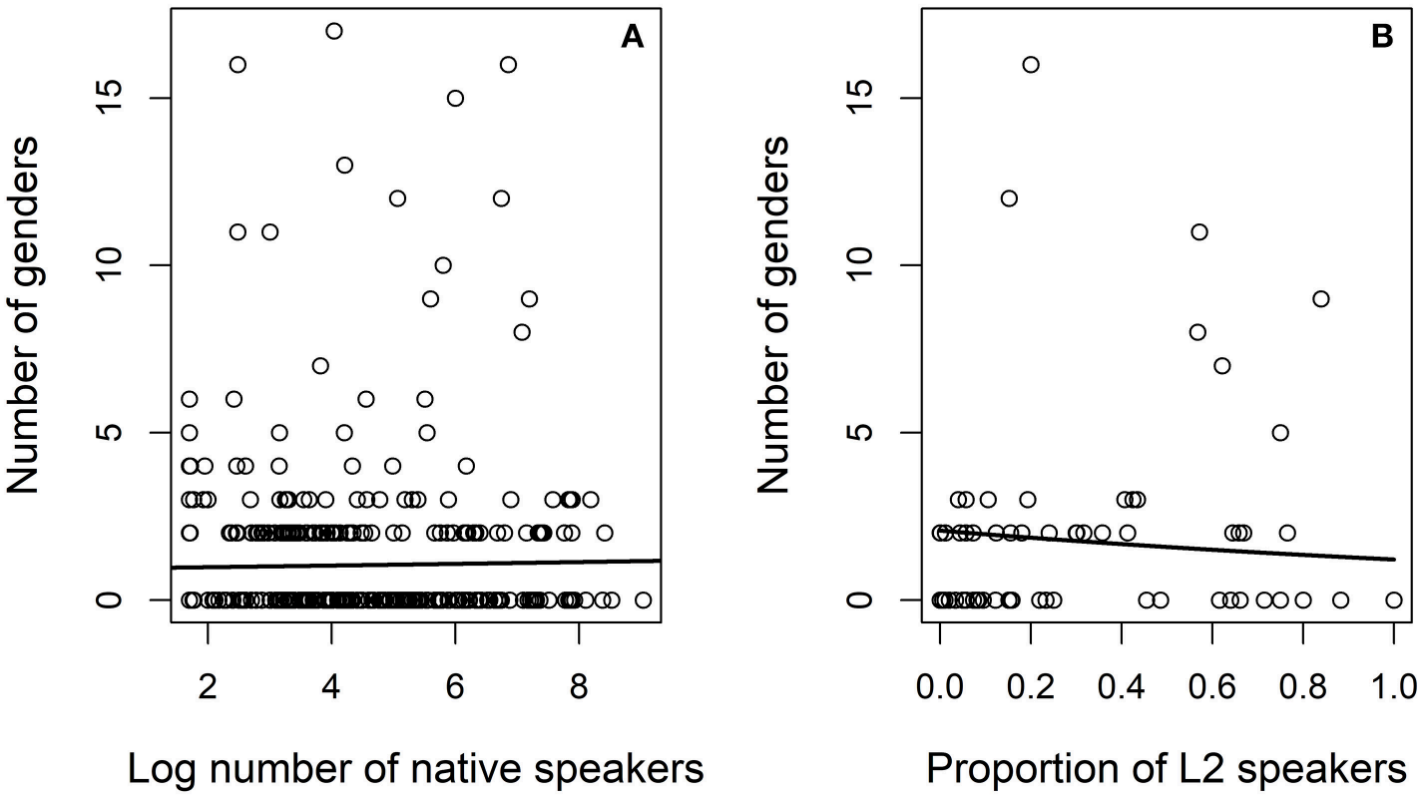
Number of genders as a function of log number of speakers on the left **(A)** and for the proportion of L2 speakers on the right **(B)**.

Figure 12 presents the number of genders as a function of the demographic variables in models GENDER.L1 and GENDER.L2. The curve indicates the fit of the mixed regression model. The figure on the left (Figure 12A) presents the fit to log number of L1 speakers. As is evident from the plot, the fitted line is almost flat. The figure on the right (Figure 12B) presents the fit to the proportion of L2 speakers. There is a small downward trend so that in communities with few L2 speakers the predicted number of genders is about three and approaching two as the percentage of L2 speakers grows closer to 100%.

According to the zero-inflated negative binomial mixed logistic regression of model GENDER.L2+, the proportion of L2 speakers (including semi-speakers) had an inverse but non-significant effect on the number of genders [log(λ) = −0.67 ± 0.46; χ^2^(1) = 1.29;*p* = 0.26]. We again tested the random effect structure with maximum likelihood ratio tests and removed the random slope for both Stocks and Area [random slope over Stocks: χ^2^(1) = 0.09;*p* = 0.76; random slope over Area: χ^2^(1) = 0.01;*p* = 0.93]. According to the reduced model the proportion of L2 speakers (including semi-speakers) had an inverse but non-significant effect on the number of genders [log(λ) = −0.59 ± 0.52; χ^2^(1) = 0.93;*p* = 0.34]. Based on this result the proportion of L2 speakers had no effect on the number of genders.

In model GENDER.ALL we model the effects of the log number of L1 speakers and the proportion of L2 speakers in competition with one another. This time we include semi-speakers for reasons of improved convergence compared to when excluding semi-speakers. According to the zero-inflated negative binomial mixed logistic regression of the maximal model, log number of L1 speakers had a non-significant inverse effect on the number of genders [log(λ) = −0.13 ± 0.21; χ^2^(1) = 0.88;*p* = 0.35]. The proportion of L2 speakers had also an inverse but non-significant effect on the number of genders [log(λ) = −0.258 ± 0.74; χ^2^(1) = 0.51;*p* = 0.48]. We again tested the random effect structure with maximum likelihood ratio tests because most of the random effect variances for the slopes (both Stocks and Area) were very close to zero (of the magnitude of 1e-7) and ended up removing all the random slopes (all were non-significant). According to the reduced model the log number of L1 speakers had a non-significant inverse effect on the number of genders [log(λ) = −0.05 ± 0.10; χ^2^(1) = 0.21;*p* = 0.65] and so did the proportion of L2 speakers [log(λ) = −0.62 ± 0.50; χ^2^(1) = 1.08;*p* = 0.30].

We further used AICc for model comparison; the results are reported in Table 7 in decreasing order of AICc. The model (4) which contained only the random intercepts but no fixed effects had the smallest AICc value (245.5). Based on the Akaike weights this model had more than 50% chance of being the best model among the four models. These results clearly suggests that neither of the demographic factors had any meaningful effect on the distribution of the number of genders.

**Table 7:** Results of model comparison for the reduced model GENDER.ALL. The full model includes both the log number of L1 speakers and the proportion of L2 speakers (including semi-speakers).

**Model**	**Model structure**	**Df**	**AICc**	**Reduction in AICc**	**Akaike weights**
(1)	Full model	5	249.2	0.0	0.080
(2)	Log number of L1 speakers	4	247.9	1.3	0.160
(3)	Proportion of L2	4	247.0	2.2	0.247
(4)	Only random structure	3	245.5	1.5	0.512

As a summary, the results of study 2 suggest that the number of L1 speakers and the proportion of L2 speakers do not have a significant effect on the number of genders. The estimate was negative for both demographic factors (except in GENDER.L1), but since the effects were non-significant and the AICc values were small, the only reliable conclusion to draw from these results is that the log number of L1 speakers and the proportion of L2 speakers have no effect on the number of genders.

#### 3.3.3. Discussion

None of the four statistical tests that we carried out to investigate the relationship between number of gender distinctions and population data yielded significant results. These (negative) findings replicate and expand on previous research by Dahl (unpublish) and suggest that the hypothesis whereby gender systems adapt to demographic variables must be rejected, at least based on the present data set.

Even though all the tests failed to reach significance, one interesting pattern emerged from the data as a function of the feature values assigned to our dependent variable “Number of genders.” We first tested whether the overall results could be affected by counting the exact number of genders for any of the sampled languages. Thereafter, we tested the relationship between the number of genders and population structure by using the classification of Corbett (2013a) in *WALS*. This classification uses five values for number of gender distinctions: “none,” “two,” “three,” “four,” “five or more.” Conflating number of genders greater than four into one bin, “five or more,” means to assume that a language with, say, 12 genders would not behave differently from a language with five genders. However, we found that using the *WALS* classification had a big impact on the results.

In particular, when we modeled the effect of the proportion of L2 speakers on number of genders and used the exact count of gender distinctions for languages with more than five genders, we found a non-significant negative correlation between the proportion of L2 and the number of genders. When following the *WALS* coding, which collapses together all languages with five or more genders, the observed coefficient between the number of genders and L2 proportions was instead positive [maximal zero-inflated negative binomial model; log(λ) = 0.84 ± 0.45; χ^2^(1) = 2.98;*p* = 0.11], even though still non-significant. This same pattern was observed when the proportion of L2 speakers also included the number of semi-speakers. The correlation coefficient was negative (but non-significant) when the exact number of genders was factored in, but it became positive (and still non-significant) when we followed the *WALS* data coding structure [maximal negative binomial model; log(λ) = 0.66 ± 0.44; χ^2^(1) = 1.61;*p* = 0.20], that is, when we lumped together languages with five or more gender distinctions.

As for the number of L1 speakers, the choice of coding had a parallel outcome. When we modeled the effect of the number of L1 speakers on the number of genders and used the exact count of gender distinctions, we found a non-significant positive correlation between the variables. When, following the *WALS* coding, we collapsed together all languages with five or more genders the observed estimate was instead negative [maximal zero-inflated poisson model; log(λ) = −0.014 ± 0.05; χ^2^(1) = 0.07;*p* = 0.79], even though still non-significant.

While these results do not affect the overall outcome of the case study, the mismatching patterns demonstrate that data structure and data coding may act as crucial confounding factors when running statistical tests on already available databases. In this particular case, the results suggest that a less abstract coding approach than the one adopted by *WALS* is preferable when investigating sociolinguistic correlates of number of gender distinctions and that the assumption we make about the behavior of languages with five or more genders matters crucially.

With regard to data coding, a parallel case reported in the literature is the correlation between phoneme inventory size and population size by Atkinson (2011). Using the *WALS* data, Atkinson (2011) arrived at a significant negative correlation between phoneme inventory and population size, which seemed to be connected to geographical spread, namely, to the spread of languages out of Africa. The *WALS* data for number of consonants divides data into five bins: “small,” “moderately small,” “average,” “moderately large,” “large.” Maddieson et al. (2011) took the underlying data for the same *WALS* chapter and still found a significant correlation, but Donohue and Nichols (2011) and Moran et al. (2012) used completely different data sets and found no significant correlation between phoneme inventory and population size reflected there. Alongside with our own results from number of genders and population size, the controversy about phoneme inventory and population data thus suggests that data, and data coding, clearly matter.

In addition, our impression is that, particularly in the case of grammatical gender, the confounding effect of data and data coding may even be a reflection of the type of variable chosen as a proxy of complexity. As outlined in section 3, recent research (Audring, 2014, 2017; Di Garbo, 2016) posits that number of gender distinctions is one of the three main dimensions of complexity variation in gender systems, along with gender assignment rules (whether gender assignment is semantic/formal, rigid/flexible), and formal marking (which word classes inflect for gender in a given language). These studies show that complexity at the level of gender distinctions predicts complexity in other domains of the gender system. For instance, Di Garbo (2016) observes that out of a sample of 84 African languages, particular instances of flexible gender assignment are only attested in languages with a high number of gender distinctions or a high degree of formal marking. Similarly, Audring (2014) observes that in languages with a high number of gender distinctions, complexity in the domain of formal marking (i.e., presence of gender marking on different types of targets in the clause) may facilitate the learning and use of gender distinctions (the more occurrences of gender marking within the utterance the easier to remember the gender of a noun). Thus, while it is no doubt that complexity in the domain of number of gender distinctions bears relevant interactions with complexities in other areas of the gender system of a language, it may well be that this type of complexity is not sensitive (or not in straightforward ways) to the effect of sociolinguistic variables. This would suggest that, in order to investigate the sociolinguistic typology of gender systems from a quantitative point of view, other typological variables than number of genders must be used. This consideration, which is also embraced by Dahl (unpublish), is the point of departure of recent research by Di Garbo and Verkerk (2017). They observe that neither the number of genders nor any of the other *WALS* variables for gender systems directly tackle the morphosyntactic encoding of gender distinctions, that is, the structural properties of gender marking systems. Under the assumption that it is morphosyntax which is directly sensitive to the effect of sociolinguistic variables, they thus look at synchronic variation in gender marking patterns in a sample of 253 Bantu languages, which are well known in the literature for their rather elaborated systems of gender marking. The study finds a significant positive correlation between incidence of restructuring in gender marking and population size whereby languages with larger populations show a preference for restructured gender marking systems^9^. This result partially contradicts the findings on creole languages by Blasi et al. (2017), who find no evidence for adaptive patterns in gender marking on adjectival modifiers and personal pronouns, the two gender-related variables included in the *APICS* database (Michaelis et al., 2013), which the study is based upon. However, while Blasi et al. (2017) only look at these two domains of gender marking, Di Garbo and Verkerk take into account a wider range of syntactic domains (adnominal modification, predication, relative constructions and pronouns) and, within each of these domains they consider different kind of gender marking hosts (for instance, within the domain of adnominal modification, they look not only at adjectival modifiers but also at numerals, demonstratives, quantifiers and question words). These results thus suggest that support to the linguistic adaptation hypothesis in the domain of grammatical gender comes from typological variables that are not (entirely) part of those typological databases that have so far been used to run exploratory studies on the relationship between language structures and social structures.

## 4. General discussion and concluding remarks

Starting from the assumption that languages are complex adaptive systems (Beckner et al., 2009), in this paper we investigated the hypothesis that morphological complexity is sensitive to sociolinguistic variables concerning population structure. This was done by means of two case studies, one in the verbal domain (degree of inflectional synthesis) and one in the nominal domain (grammatical gender). In both case studies, the same type of sociolinguistic data were operationalized as independent variables: population size (measured as log number of L1 speakers) and proportion of L2 speakers (including/excluding semi-speakers in different models). The raw data for the typological variables came from the *AUTOTYP* database for inflectional synthesis on the verb and from Sinnemäki (unpublish) and *WALS* (Corbett, 2013a) for grammatical gender. The raw demographic data were taken mostly from the *Ethnologue* (see the Supporting Material). While the results of case study 1 confirm that morphological complexity in the verbal domain is sensitive to population dynamics thus bringing support to the main hypothesis, the same could not be observed in the case of grammatical gender (case study 2).

However, irrespectively of how well the individual case studies support the main hypothesis, we think that both make a relevant contribution to the understanding of non-linguistic correlates of linguistic diversity. First, the results of the two case studies suggest that not all domains of grammar adapt to sociolinguistic variables to the same extent. More specifically, our data show that while the degree of inflectional synthesis is sensitive to population data, the number of gender distinctions is not. Whether this discrepancy is related to the different functions that the two grammatical domains display in discourse is an open question whose answer we leave to further studies. Our results ultimately suggest that no general prediction can be made about the relationship between morphological complexity and population data because the outcomes of this relationship are very much specific to the grammatical domain under study.

Second, the results from study 1 suggest that competitive models, where the effect of multiple sociolinguistic variables on language structures are tested simultaneously, are somewhat better than non-competitive models, where each factor is tested in isolation. These findings bring quantitative evidence in support of Trudgill's (2011a,b) suggestion that the effect of social structures on language structures must be studied by factoring in a multifaceted array of interacting variables, ranging from population size to degree of contact and social network density. While our study covers two of the three suggested dimensions—population size and degree of contact (of which the proportion of L2 speakers is taken as a proxy)—nothing could be said about social network density. Operationalizing social network density as one of the critical variables in quantitative sociolinguistic typology would, in fact, require accessing a type of data that is at present not featured in existing databases.

Third, in line with previous studies addressing similar research questions, case study 2 fails to show any significant relationship between the complexity of grammatical gender systems (measured in terms of number of gender distinctions) and sociolinguistic variables. These results contradict the well-known observation (supported by evidence from different linguistic families and areas) that while gender systems are generally very stable, their transmission tends to be disrupted under the pressure of language contact. In line with a recent suggestion by Dahl (unpublish) and ongoing research on the topic (Di Garbo and Verkerk, 2017) we think that a reasonable explanation behind this mismatch may be that the number of gender distinctions is not a suitable measure to test hypotheses on linguistic adaptation in the domain of grammatical gender, and that typological variables pertaining to patterns of gender marking should instead be considered. In addition, we found that using the number of gender distinctions as coded in *WALS*, with five cut-off points between no gender, two, three, four, five or more gender distinctions, leads to less accurate results than following a less abstract coding procedure where languages with richer gender systems are coded based on the exact number of distinctions that they display. For these reasons, we conclude that existing typological databases are not fully equipped to support quantitative sociolinguistic typologies of grammatical gender systems.

To sum up, while at least for one of the grammatical domains used as test cases this paper confirms the validity of the linguistic adaptation hypothesis, the paper also shows that a precondition to the advancement of research on nonlinguistic correlates of linguistic diversity lies in the refinement of the statistical methodologies used to test this hypothesis as well as in the types of data and data coding principles that are fed into the analyses. In order to test hypotheses about sociolinguistic typology, comparative data on sociolinguistic variables other than demographic variables, such as relative prestige, literacy, and multilingualism, need to be collected. Furthermore, given that approaching linguistic structures (and their complexity) from different perspectives may produce radically different results about adaptation, more exploratory studies need to be run in order to test which domains of grammar and what types of language structures within a given domain are most sensitive to the effect of social structures.

## Author contributions

The research was designed together by KS and FD, data collection was done primarily by KS (data on the number of genders for languages with 5+ genders was collected together by FD and KS), statistical analyses for the typological case studies were done by KS, and the write-up was done together by KS and FD.

### Conflict of interest statement

The authors declare that the research was conducted in the absence of any commercial or financial relationships that could be construed as a potential conflict of interest.

## Footnotes

^1^Naturally, however, a number of factors may interfere with the success rate of non-native acquisition of gender, such as the presence of a gender system in the L1, typological similarities between L1 and L2, age of acquisition, motivation.

^2^These are: “Number of gender values” (Corbett, 2013a), “Sex-based and Non-Sex-Based Gender” (Corbett, 2013b), and “Systems of gender assignment” (Corbett, 2013c).

^3^The Ethnologue Global Dataset is available at https://www.ethnologue.com/data-consulting.

^4^In glmmADMB parameters are estimated by maximum likelihood ratio using Laplace approximation. We improved this Laplace approximation by using importance sampling, providing the argument impSamp with values >0; (Skaug and Fournier, 2006).

^5^We initially evaluated the predictive capacity of our models by using the index of concordance (C) between the predicted probability and the observed response, which is quite widely used in linguistics. For the reduced model the value of C was 0.74 (values of C between 0.7 and 0.8 are considered acceptable by Hosmer and Lemeshow, 2000, p. 162). However, a reviewer pointed out that this good index of concordance might be misleading, as its value might be only due to the random structure. To double-check this, we compared the C in models with and without the fixed effect. Since the difference of these models was only 0.006, this seemed to suggest that the log number of L1 speakers has virtually no predictive power. For two reasons, we think that this conclusion would be premature. First, Barth and Kapatsinski (2018) present results of a simulation, which shows that a real predictor may fail to contribute to a model's predictive capacity measured, for instance, by C. Their result suggests that perhaps the index of concordance should not be used to assess the predictive capacity of GLMMs. Second, the predictive capacity of models can also be evaluated using *R*^2^, which measures the variance explained by the model. Although there is no consensus as to how or whether it would be possible to reliably compute *R*^2^ for GLMMs, many researchers currently use marginal *R*^2^ to compute the variance explained by the fixed effects only and conditional *R*^2^ to compute the variance explained by the whole model (both fixed and random effects) (following Nakagawa and Schielzeth, 2013; Johnson and O'Hara, 2014). Marginal and conditional *R*^2^ can be computed using the R package MuMIn (Barton, 2018), but unfortunately this is not yet implemented for models produced with glmmADMB, which we used for modeling. For this purpose, we used Bayesian mixed effects modeling with R package blme (Chung et al., 2013) to build model SYNTHESIS.L1 (our reduced model that included only the random intercepts), as it produces objects that MuMIn understands but also because the models actually converged, unlike when using the package lme. The results produced by blme[log(λ) = −0.079 ± 0.018; χ^2^(1) = 17.9;*p* = 0.000023] were practically identical compared to those produced by glmmADMB [log(λ) = −0.078 ± 0.018; χ^2^(1) = 17.8;*p* = 0.000024]. Based on the model produced by blme the marginal *R*^2^ = 0.094 and the conditional *R*^2^ = 0.279. The marginal *R*^2^ suggests that the log number of L1 speakers has reasonable predictive power, as it explains almost 10% of variance in the degree of synthesis. In addition, the conditional *R*^2^ is similar to what we have often witnessed for typological data (e.g., Sinnemäki, 2010).

^6^Created using R package effects (Fox, 2003).

^7^The AICc values as well as the Akaike weights were computed using package MuMIn (Barton, 2018).

^8^Although our data is relatively small we also tested whether the interaction term between the log number of L1 speakers and the proportion of L2 speakers would have a significant effect on the degree of inflectional synthesis. It is possible that in very large languages the population size of L1 would act as a buffer against transfer effects from the L2 population (cf. our discussion in section 3.1). For this purpose we compared a model that included this interaction term to one that excluded it (using only random intercepts for both Stock and Area). Based on the result the interaction term had a negative but non-significant effect on the degree of inflectional synthesis [log(λ) = −0.085 ± 0.082; χ^2^(1) = 1.1;*p* = 0.30]. This suggests that population size and the proportion of L2 speakers influence degree of inflectional synthesis independently of one another.

^9^In this study, restructured gender systems are systems in which gender marking is partially or heavily based on animacy distinctions
