# Supplementary material for: Language Structures May Adapt to the Sociolinguistic Environment, but It Matters What and How You Count: A Typological Study of Verbal and Nominal Complexity
Source: Front Psychol. 2018 Aug 14;9:1141. doi: 10.3389/fpsyg.2018.01141 (PMC6102949; doi:10.3389/fpsyg.2018.01141)
Supplement: Supplementary file 1 [file Presentation_1.pdf]

Supplementary material:

Language structures may adapt to the sociolinguistic environment, but it matters what and how you count: A typological study of verbal and nominal complexity

Kaius Sinnemäki and Francesca Di Garbo

Description of the dataset for typological study 1

We provide here the description of the data and sources for case study 1. The full dataset (Data Sheet 1.XLSX) provides metadata for the sample languages and the data on degree of verbal inflectional synthesis and on the demographic variables used in the case study. The following list describes each datatype in the tab called "Data" in the Excel file.

Tab "Data"

- Column A: Language name (as in *AUTOTYP*; Bickel et al. 2017)
- Column B: ISO639.3 code (as in the 19th edition of the *Ethnologue*; Lewis et al. 2016)
- Column C: Glottolog code (Hammarström et al. 2017)
- Column D: Stock name (This is the highest level of classification in the genealogical classification of the *AUTOTYP*; Bickel et al. 2017)
- Columns E and F: The longitudinal and latitudinal data (as in *AUTOTYP*; Bickel et al. 2017)
- Column G: The geographical continent in which the language is spoken (*AUTOTYP*; Bickel et al. 2017). The *AUTOTYP* divides the world into ten continents.
- Column H: The geographical area in which the language is spoken (*AUTOTYP*; Bickel et al. 2017). The *AUTOTYP* divides the world into 24 areas.
- Column I: Data on the degree of inflectional synthesis of the verb (from the column "VInflCatAndAgrMax.n" in the *AUTOTYP* file Synthesis.csv; Bickel et al. 2017)
- Column J: The number of native speakers. The number is for "all countries", not just for the main country where the language is spoken. The data is from the 19th edition of the *Ethnologue* (Lewis et al. 2016) if not otherwise specified by superscripts (a-o) in Column K.
- Column K: The source for the number of native speakers. E19 means the 19th edition of the *Ethnologue*. The reference of the sources is specified in the tab "References" of the Excel file.
- Column L: The number of second language speakers. The reference of the sources is specified in the tab "References" of the Excel file. The entry "NA" means that no information or no reliable information was available for that language.
- Column M: The source for the number of second language speakers. The reference of the sources is specified in the tab "References" of the Excel file.
- Column N: The number of semi-speakers. The source in all cases is the 19th edition of the *Ethnologue*. The entry "NA" means that no information was available.

Tab "References"

- Column A: The reference for the source.

Description of the dataset for typological study 2

We provide here the description of the data and sources for case study 2. The full dataset (Data Sheet 2.XLSX) provides metadata for the sample languages and the data on the number of genders and on the demographic variables used in the case study. The following list describes each datatype in the tab called "Data" in the Excel file.

Tab "Data"

- Column A: Language name (as in *AUTOTYP*; Bickel et al. 2017)
- Column B: ISO639.3 code (as in the 19th edition of the *Ethnologue*; Lewis et al. 2016)
- Column C: Glottolog code (Hammarström et al. 2017)
- Column D: Stock name (This is the highest level of classification in the genealogical classification of the *AUTOTYP*; Bickel et al. 2017)
- Columns E and F: The longitudinal and latitudinal data (as in *AUTOTYP*; Bickel et al. 2017)
- Column G: The geographical continent in which the language is spoken (*AUTOTYP*; Bickel et al. 2017). The *AUTOTYP* divides the world into ten continents.
- Column H: The geographical area in which the language is spoken (*AUTOTYP*; Bickel et al. 2017). The *AUTOTYP* divides the world into 24 areas.
- Column I: The number of genders. The data is largely taken from Sinnemäki (forthcoming) and Corbett (2013).
- Columns J-K: The sources for the number of genders. The references are specified in the tab "ReferencesGender" of the Excel file.
- Column L: The number of native speakers. The number is for "all countries", not just for the main country where the language is spoken. The data is from the 19th edition of the *Ethnologue* (Lewis et al. 2016) if not otherwise specified in Column M.
- Column M: The source for the number of native speakers. E19 means the 19th edition of the *Ethnologue*. The references are specified in the tab "ReferencesDemog" of the Excel file.
- Column N: The number of second language speakers. The data is from the 19th edition of the *Ethnologue* (Lewis et al. 2016) if not otherwise specified in Column O. The entry "NA" means that no information or no reliable information was available for that language.
- Column O: The source for the number of second language speakers. E19 means the 19th edition of the *Ethnologue*. The references are specified in the tab "ReferencesDemog" of the Excel file.
- Column P: The number of semi-speakers. The source in all cases is the 19th edition of the *Ethnologue*. The entry "NA" means that no information or no reliable information was available for that language.

Tab "ReferencesGender"

- Column A: The reference for the source.

Tab "ReferencesDemog"

- Column A: The reference for the source.
